# Engineered Exosome‐Based Senolytic Therapy Alleviates Stroke by Targeting p21^+^CD86^+^ Microglia

**DOI:** 10.1002/EXP.20240349

**Published:** 2025-03-06

**Authors:** Jialei Yang, Shipo Wu, Miao He

**Affiliations:** ^1^ Department of Neurology, China National Clinical Research Center for Neurological Diseases Beijing Tiantan Hospital, Capital Medical University Beijing China; ^2^ Laboratory of Advanced Biotechnology Beijing Institute of Biotechnology Beijing China

**Keywords:** engineered exosomes, extracellular vesicles, ischemic stroke, microglia, senescence, targeted delivery

## Abstract

Stroke remains the leading cause of neurological mortality and disability worldwide, with post‐stroke inflammation significantly hindering neural repair. Despite its critical impact, mechanism‐based therapeutic strategies are scarce. In this study, we uncovered a critically important yet previously unexamined cell population, p21^+^CD86^+^ microglia, which accumulated in ischemic region. Unexpectedly, we discovered that p21 interacted with C/EBPβ, driving C/EBPβ‐dependent transcription and upregulating key pro‐inflammatory factors such as *Il6*, *Il1β*, *Cxcl2*, and *Cxcl10*. To specifically target and eliminate these pathogenic p21^+^CD86^+^ microglia, we engineered exosomes with a peptide that selectively binds CD86^+^ microglia and loaded them with the senolytic Quercetin. Furthermore, we developed an optimized, stable Que@micro‐Exo therapeutic formulation. Systemic administration of Que@micro‐Exo robustly reduced p21^+^CD86^+^ microglia and suppressed their pro‐inflammatory phenotype. Notably, functional analyses revealed that Que@micro‐Exo treatment mitigated blood‐brain barrier disruption, promoted beneficial microglial polarization, decreased neutrophil infiltration, and significantly enhanced functional recovery following cerebral ischemia, all with a favorable safety profile. Our preclinical findings lay the foundation for targeting p21^+^CD86^+^ microglia as a novel therapeutic strategy, highlighting the potential of exosome‐based senolytic anti‐inflammatory therapy for stroke and other central nervous system disorders.

## Graphical abstract

We identified a harmful immune cell type, p21^+^CD86^+^ microglia, driving post‐stroke inflammation and developed a targeted exosome‐based therapy (Que@micro‐Exo) to eliminate these cells. This innovative treatment reduced inflammation, alleviated brain damage, and improved recovery in stroke models, offering a promising strategy for stroke and other central nervous system disorders.

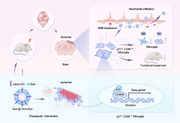

## 1 Introduction

Stroke is the leading cause of neurological mortality and disability worldwide, profoundly impacting millions of lives and placing substantial burdens on healthcare systems. Among all stroke types, ischemic strokes account for 62.4% to 87.0% of cases [1]. The aftermath of ischemic stroke is characterized by intense neuroinflammation, leading to blood‐brain barrier (BBB) breakdown, microglial activation, and the infiltration of peripheral immune cells, all of which exacerbate brain damage and worsen neurological outcomes [2]. Despite the critical need, effective mechanism‐based therapies remain scarce, highlighting the necessity for novel strategies to mitigate these detrimental effects and promote recovery.

Cellular senescence is a distinct cell state characterized by permanent cell cycle arrest, typically triggered by cellular stress or damage. This state is driven by cyclin‐dependent kinase inhibitors such as p21 and p16 and is often accompanied by the senescence‐associated secretory phenotype (SASP)—a distinct profile of pro‐inflammatory cytokines, chemokines, and proteases that can exacerbate tissue damage [3]. While cellular senescence can serve protective roles in specific physiological contexts, such as wound healing and embryogenesis, its effects are predominantly harmful under pathological conditions. Accumulating evidence has demonstrated the contribution of senescence and SASP in aging, traumatic brain injury, and neurodegenerative disorders such as Alzheimer's disease [4]. These findings suggest that senescence‐related mechanisms may also be relevant to the pathology of ischemic stroke. However, the specific roles of cellular senescence in ischemic stroke, particularly regarding specific cell subpopulations, cellular phenotypes, and underlying mechanisms, remain poorly understood. Bridging this knowledge gap is critical to identifying potential therapeutic strategies that target senescence to improve outcomes after ischemic stroke.

The clearance of senescent cells is an emerging therapeutic approach to mitigate their deleterious effects. Senolytic agents, such as quercetin, are designed to selectively eliminate senescent cells and alleviate senescence‐associated pathologies. Quercetin, a natural plant‐derived flavonoid, has demonstrated senolytic activity in various diseases [5]. Beyond its anti‐senescence properties, quercetin exhibits pleiotropic effects, including anti‐inflammatory, anti‐oxidative, and neuroprotective activities. However, quercetin's clinical applicability is hindered by its water insolubility, low bioavailability due to extensive first‐pass metabolism, and lack of specificity in targeting senescent cell subtypes. Additionally, its inability to effectively cross the BBB poses a significant challenge for its use in treating central nervous system (CNS) diseases.

Recent evidence has demonstrated that exosomes, extracellular vesicles sized 30–150 nm, offer a promising solution for drug delivery challenges due to their unique properties [6, 7]. Exosomes are naturally derived from cells and play a critical role in intercellular communication, making them inherently biocompatible and less immunogenic compared to synthetic carriers like lipid nanoparticles. Exosomes exhibit superior capability to cross biological barriers, including the blood‐brain barrier (BBB), and can be engineered for targeted delivery, enhancing their therapeutic potential [8]. Our previous work demonstrated the fusion of rabies virus glycoprotein (RVG) with the exosomal protein Lamp2b, enabling engineered exosomes to deliver therapeutic molecules such as microRNA‐124, recombinant human NGF protein, and its mRNA into the brain [9, 10]. These advancements led to a clinical trial based on our preclinical study [11]. Building on this success, we propose that using engineered exosomes as delivery vehicles for senolytic agents like quercetin could address critical challenges such as BBB crossing, cell‐specific targeting, and drug solubility. Furthermore, their natural origin and ability to be personalized for specific therapeutic applications further underscore their potential advantages over other nano‐delivery systems.

In this study, we have identified a previously unexamined population of senescent cells: p21^+^CD86^+^ microglia. While p21 is a well‐known marker of cellular senescence, the specific role of p21‐expressing microglia in the context of ischemic brain injury remains largely unexplored. Our research elucidates the mechanism underlying p21‐mediated, CCAAT/enhancer‐binding protein beta (C/EBPβ)‐dependent SASP in these microglia. By employing bioengineering technology, we developed an exosome‐based senolytic therapy (Que@micro‐Exo) and evaluated its safety and efficacy for stroke treatment. Our findings highlight the causal role of p21^+^CD86^+^ microglia in neuroinflammation and uncover novel therapeutic targets to enhance recovery in stroke patients. Furthermore, our preclinical study suggests that exosome‐based senolytic therapy could have broader clinical applications for other central nervous system diseases.

## 2 RESULTS

### 2.1 p21^+^CD86^+^ Microglia Accumulate in Ischemic Regions

**FIGURE 1:**
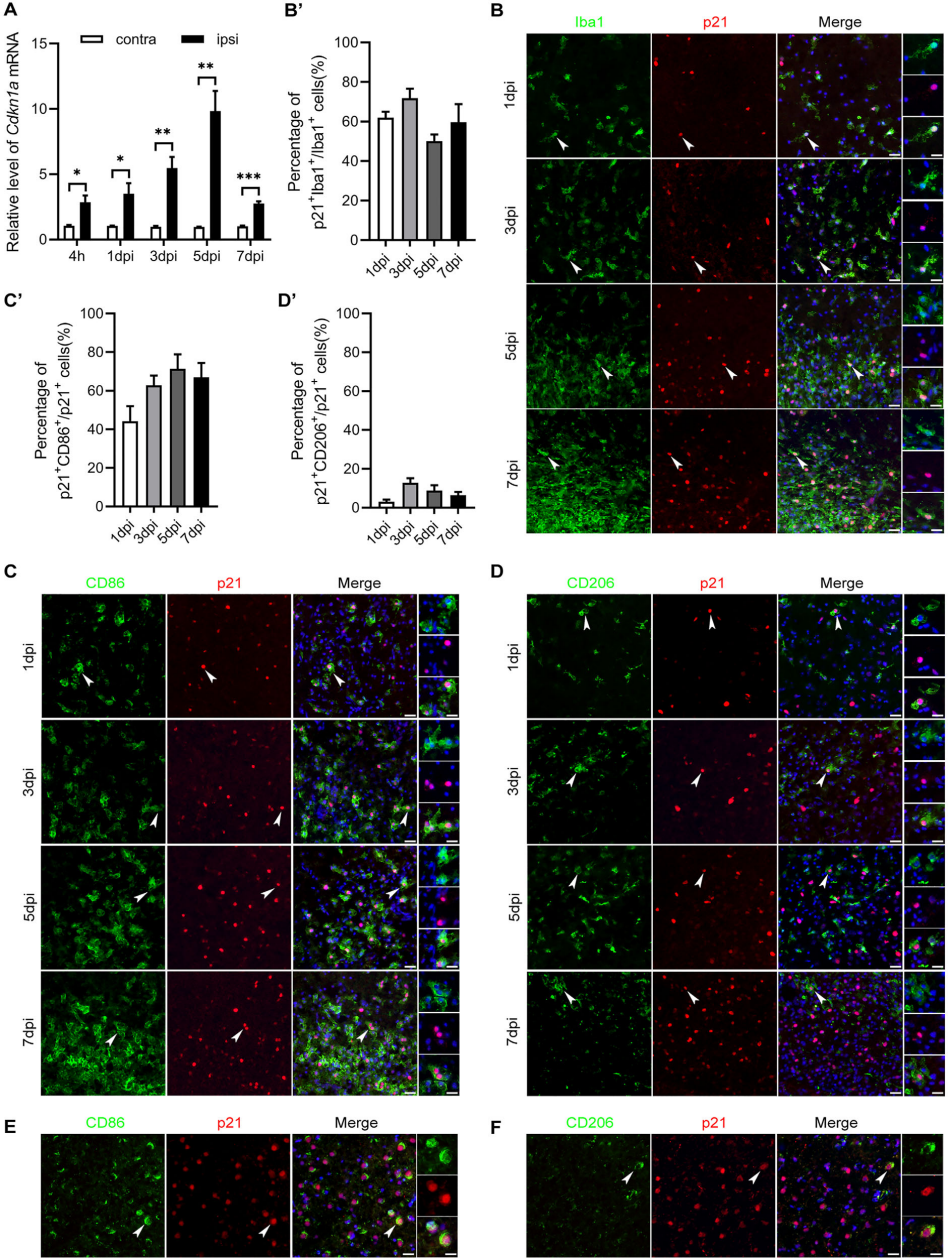
p21^+^CD86^+^ microglia accumulate in ischemic regions. (A) *Cdkn1a* mRNA levels in contralateral (contra) and ipsilateral (ipsi) regions at 1, 3, 5, and 7 days post‐ischemia (dpi). (B–D) Representative immunofluorescence images of Iba1/p21, CD86/p21, and CD206/p21. Note the prominent colocalization of Iba1/p21 and CD86/p21, indicating the significant accumulation of p21^+^CD86^+^ microglia. Arrows highlight representative double‐positive cells; the cell indicated by the arrow is magnified. Scale bar = 30 µm; bars = 20 µm in the magnified images. (B’–D’) Quantification of the percentage of double‐positive cells in panels B–D. (E,F) Representative immunofluorescence images of CD86/p21 and CD206/p21 in the ischemic cortical region of stroke patients. Scale bar = 30 µm. Data are expressed as mean ± SEM, with *n* = 3 per group. Data were analyzed by an unpaired two‐tailed Student's *t*‐test. *p < 0.05; **p < 0.01; ***P < 0.001.

To elucidate the expression patterns of the senescence markers p21 (coded by *Cdkn1a*) and p16 (coded by *Cdkn2a*) in ischemic brain, we employed a photothrombotic ischemia model in mice. qRT‐PCR analysis showed a significant increase in *Cdkn1a* expression as early as 4 h post‐ischemia, with a steady rise at 1, 3, 5, and 7 days post‐ischemia (dpi), peaking at 5 dpi (Figure 1A). In contrast, p16 expression exhibited a modest increase at 4 h and 1 dpi, becoming significant at 3, 5, and 7 dpi, but to a lesser extent than p21 (Figure S1A). These results indicate that p21 is an earlier and more prominent senescence marker in ischemic regions compared to p16.

Immunofluorescent staining of microglial marker Iba1 with p21 or p16 revealed that 50.12%–71.80% of Iba1‐positive cells were also positive for p21 (Figure 1B,B’), whereas only 0.66%–3.11% were positive for p16 (Figure S1B). This demonstrates that p21 is a more dominant marker than p16 in microglia following ischemia. To further investigate the phenotypic distribution of M1 and M2 microglia among p21‐positive cells, we assessed the expression of CD86 and CD206, classic markers for M1 and M2 microglia, respectively. The results showed that CD86/p21 cells accounted for 44.24%–71.40% (Figure 1C,C’), while CD206/p21 cells accounted for 3.15%–12.84% (Figure 1D,D’). This indicates that CD86‐positive M1 microglia predominate among p21‐positive microglia in ischemic regions.

To verify whether this phenomenon occurs in humans, we examined cortical samples from ischemic stroke patients 24 h post‐ischemia. Immunofluorescence analysis revealed that p21‐positive cells predominantly expressed the M1 microglia marker CD86 (Figure 1E), with few co‐labeling of the M2 marker CD206 (Figure 1F). This finding indicates that p21^+^CD86^+^ microglia are also present in the human brain post‐ischemia. Our results identify p21 as a key early and predominant senescence marker in ischemic brain regions, particularly within microglia. The predominance of p21^+^CD86^+^ M1 microglia in both mice and human ischemic brains highlights their potential role in the inflammatory response following ischemic stroke.

### 2.2 p21^+^CD86^+^ Microglia Exhibit a Dominant Pro‐Inflammatory Phenotype

**FIGURE 2:**
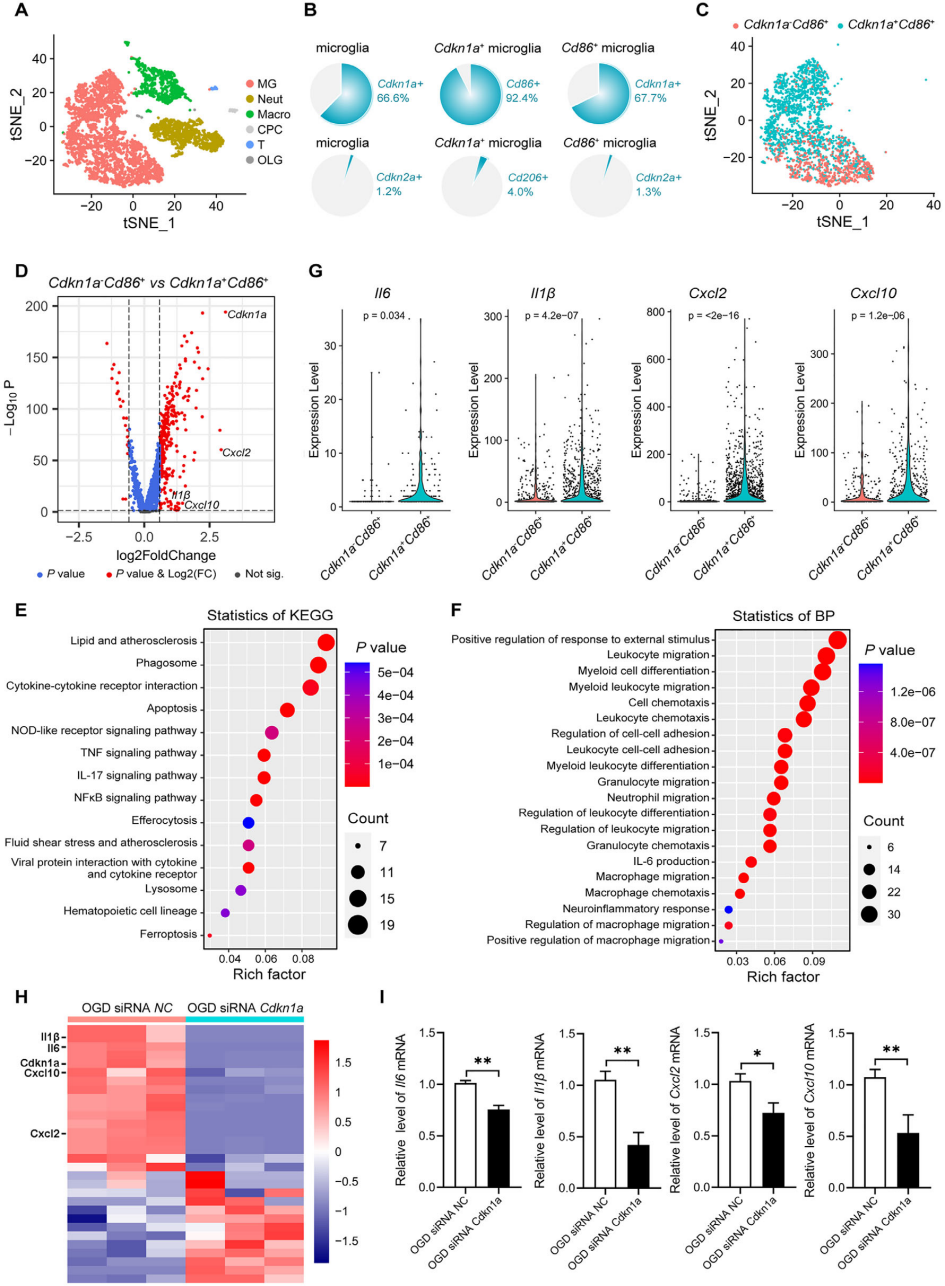
p21^+^CD86^+^ microglia exhibit a dominant pro‐inflammatory phenotype. (A) Single‐cell RNA sequencing t‐SNE plot displaying the clusters and annotations of cells. MG, microglia; Neut, neutrophils; Macro, macrophages; CPC, choroid plexus cells; T, T cells; OLG, oligodendrocytes. (B) Pie charts showing the proportions of microglia subpopulations. (C) t‐SNE plot illustrating the clusters of *Cdkn1a* ^−^ *Cd86* ^+^ and *Cdkn1a* ^+^ *Cd86* ^+^ cells. (D) Volcano plot illustrating the differential gene expression. *X*‐axis: log2 fold change (log2FC). *Y*‐axis: −log10 *p*‐value. Significance thresholds: vertical dashed lines at log2FC = ±1.5; horizontal dashed line at *p* = 0.05. (E,F) Kyoto Encyclopedia of Genes and Genomes (KEGG) pathway and Gene Ontology (GO) biological process (BP) enrichment analysis of differentially expressed genes. (G) Violin plots illustrating the expression levels of *Il6*, *Il1β*, *Cxcl2*, and *Cxcl10*. (H) Heat map showing the bulk RNA sequencing of OGD BV2 cells treated with siRNA NC and siRNA *Cdkn1a*. *n* = 3 per group. (I) qRT‐PCR analysis showing the expression levels of *Il6*, *Il1β*, *Cxcl2*, and *Cxcl10* in OGD BV2 cells treated with siRNA NC and siRNA *Cdkn1a*. Data are expressed as mean ± SEM, with *n* = 3 per group. Data were analyzed by an unpaired two‐tailed Student's *t*‐test. *p < 0.05; **P < 0.01.

To gain a deeper understanding of p21^+^CD86^+^ microglia in the ischemic region, we analyzed the single‐cell RNA sequencing (scRNA‐seq) data of *Cd45*‐positive cells in this area at 1dpi. Using unsupervised clustering and the expression of cell‐type‐specific markers [12], we identified distinct clusters of microglia (*Fcrls, Cx3cr1*), macrophage (*Lyz2, S100a4*), neutrophil (*Retnlg, Hdc*), T cells (*Trbc2, Cd3d*), choroid plexus cells (*Htr2c, Slc4a5*), and oligodendrocytes (*Plp1, Mog*) (Figure 2A and Figure S2). Analysis of pie charts revealed that 66.6% of microglia were *Cdkn1a* (p21) positive, while only 1.2% were positive for *Cdkn2a* (p16) (Figure 2B). Among the *Cdkn1a*‐positive microglia, a significant majority (92.4%) were *Cd86* positive, indicating a strong pro‐inflammatory phenotype. In contrast, only 4.0% of *Cdkn1a*‐positive microglia expressed the anti‐inflammatory marker *Cd206*. Additionally, among *Cd86*‐positive microglia, 67.7% expressed *Cdkn1a*, and a mere 1.3% expressed *Cdkn2a*. These findings demonstrate that a significant majority of microglia in the ischemic region express *Cdkn1a* and are predominantly associated with a pro‐inflammatory phenotype, characterized by high *Cd86* expression.

We next compared the transcriptomes between *Cdkn1a* ^−^ *Cd86* ^+^ and *Cdkn1a* ^+^ *Cd86* ^+^ cells, identifying 354 genes upregulated (log2 fold change > 1.5) and 23 genes downregulated (log2 fold change < −1.5) in *Cdkn1a* ^+^ *Cd86* ^+^ cells (*p* value < 0.05) (Figure 2C,D). Kyoto Encyclopedia of Genes and Genomes (KEGG) pathway and gene ontology (GO) biological process (BP) analysis of upregulated genes revealed that *Cdkn1a* ^+^ *Cd86* ^+^ cells exhibited higher activity in inflammatory response and chemotaxis (Figure 2E,F). Specifically, four pro‐inflammatory factors, *Il6*, *Il1β*, *Cxcl2*, and *Cxcl10*, commonly associated with the SASP, were significantly upregulated in *Cdkn1a* ^+^ *Cd86* ^+^ cells compared to *Cdkn1a* ^−^ *Cd86* ^+^ cells (Figure 2G). This indicates that while both populations were Cd86^+^ M1 microglia, *Cdkn1a* ^+^ *Cd86* ^+^ cells exhibit a more deleterious pro‐inflammatory phenotype, suggesting a critical role for *Cdkn1a* (p21) in promoting inflammation.

To further investigate the role of p21 in inflammation, we designed and tested siRNAs targeting *Cdkn1a* (Figure S3). Using a BV2 microglia OGD model, we transfected cells with siRNA *Cdkn1a* and performed bulk RNA sequencing. This revealed significant downregulation of *Cdkn1a*, *Il6*, *Il1β*, *Cxcl2*, and *Cxcl10* expression in the siRNA *Cdkn1a* group (Figure 2H). qRT‐PCR further confirmed these results (Figure 2I). Our findings demonstrate that *Cdkn1a* (p21) significantly contributes to the M1 pro‐inflammatory phenotype of microglia, offering potential therapeutic targets for mitigating neuroinflammation in stroke.

### 2.3 p21 Mediates Inflammatory Phenotype by Interacting With C/EBPβ

**FIGURE 3:**
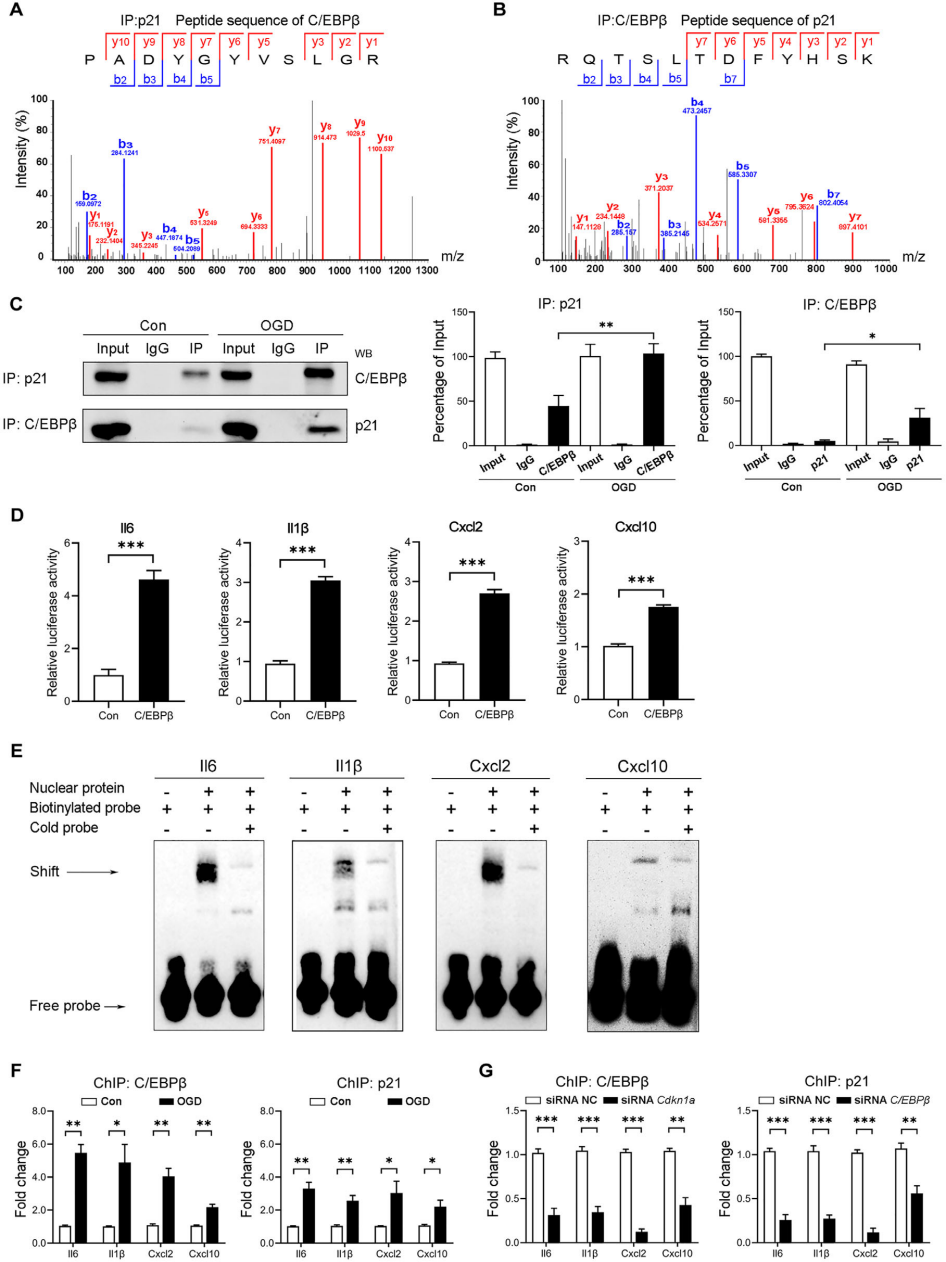
p21 mediates inflammatory phenotype by interacting with C/EBPβ. (A,B) Mass spectrometry analysis showing the characteristic peptide sequence of C/EBPβ and p21. *X*‐axis (*m*/*z*): Mass‐to‐charge ratio of detected ions. *Y*‐axis (Intensity %): Relative intensity of detected ions. (C) Immunoblotting and quantification of coimmunoprecipitation samples from BV2 cells under control (Con) and oxygen‐glucose deprivation (OGD) conditions. Data are presented as percentage of input. (D) Luciferase assay showing the relative luciferase activity in HEK293 cells transfected with overexpression plasmids for C/EBPβ and promoter reporter plasmids for *Il6*, *Il1β*, *Cxcl2*, and *Cxcl10*. (E) Electrophoretic mobility shift assay (EMSA) demonstrating the binding interaction between C/EBPβ with the *Il6*, *Il1β*, *Cxcl2*, and *Cxcl10* promoter. (F) Quantitative ChIP assay of control and OGD BV2 cells immunoprecipitated with anti‐C/EBPβ or anti‐p21 antibodies. (G) Quantitative ChIP assay of OGD BV2 cells treated with siRNA NC, siRNA C/EBPβ, or siRNA Cdkn1a. Data are expressed as mean ± SEM, with *n* = 3 per group. Data were analyzed by an unpaired two‐tailed Student's *t*‐test. *p < 0.05; **p < 0.01; ***P < 0.001.

To elucidate the mechanism through which p21 exerts its function in inflammation, we extracted nuclear fractions from BV2 cells and conducted coimmunoprecipitation with a p21 antibody. Mass spectrometry identified the transcription factor C/EBPβ, a member of the CCAAT/enhancer binding protein (C/EBP) family, as a binding partner (Figure 3A). This interaction was validated by reciprocal immunoprecipitation using a C/EBPβ antibody, confirming the p21‐C/EBPβ complex (Figure 3B). Immunoblotting of coimmunoprecipitation samples revealed significantly increased p21‐C/EBPβ binding under OGD conditions compared to controls (Figure 3C).

Given C/EBPβ’s role in driving inflammatory responses in neurodegenerative diseases and brain injury by binding to the promoter regions of cytokines and other pro‐inflammatory genes [13], we hypothesized that p21 facilitates an inflammatory phenotype through C/EBPβ‐dependent SASP release. To test this hypothesis, we employed a dual luciferase assay to evaluate C/EBPβ binding to putative motifs in the promoter regions of *Il6*, *Il1β*, *Cxcl2*, and *Cxcl10*. Bioinformatics analysis with LASAGNA‐Search 2.0 and JASPAR 2020 predicted binding sites within 2000 bp upstream of the transcription start site (TSS) [14, 15]. We then constructed pGL3 luciferase reporter plasmids encoding various truncated promoter regions. The luciferase assay indicated high transcriptional activity for *Il6* (−450 bp to TSS), *Il1β* (−501 bp to TSS), *Cxcl2* (−699 to −567 bp), and *Cxcl10* (−366 to −252 bp) under C/EBPβ regulation (Figure 3D and Figure S4A,B).

To further confirm C/EBPβ binding, we performed an electrophoretic mobility shift assay (EMSA) with biotinylated probes designed from the identified target regions (Figure 3E). The EMSA results showed that these probes bound to C/EBPβ in the HEK293 nuclear fraction, and the binding specificity was confirmed by a competition assay using cold probes, which decreased the intensity of the shifted bands.

In a quantitative ChIP assay, OGD treatment significantly enhanced the enrichment of C/EBPβ at the promoters of *Il6*, *Il1β*, *Cxcl2*, and *Cxcl10*. Although direct binding sites for p21 at these promoter regions were not found, ChIP using a p21 antibody also showed enrichment, likely due to its interaction with C/EBPβ (Figure 3F). Importantly, siRNA‐mediated knockdown of *Cdkn1a* or *C/EBPβ* significantly reduced the OGD‐induced promoter enrichment of these inflammatory factors (Figure 3G and Figure S5). Collectively, these findings suggest that p21 is crucial in regulating C/EBPβ‐mediated expression of inflammatory factors, highlighting its potential role in neuroinflammatory responses.

### 2.4 Exosome‐Based Senolytic Anti‐Inflammatory Therapy: Construction of Que@Micro‐Exo Agent

To selectively eliminate p21^+^CD86^+^ microglia, we engineered exosomes with a targeting peptide for CD86^+^ microglia and loaded them with the senolytic quercetin, thus creating quercetin‐loaded microglia‐targeted exosomes (Que@micro‐Exo). This approach aims to leverage the advantages of targeted‐delivery exosomes and the anti‐senescent properties of quercetin. Homing peptides targeting CD86‐positive M1 microglia (abbreviated as micro‐CH) have been shown to be selectively taken up by CD86‐positive M1 microglia in vitro. The high affinity and specificity enable it to target CD86‐positive M1 microglia in neuropathic pain mice, demonstrating its in vivo targeting capability [16].

**FIGURE 4:**
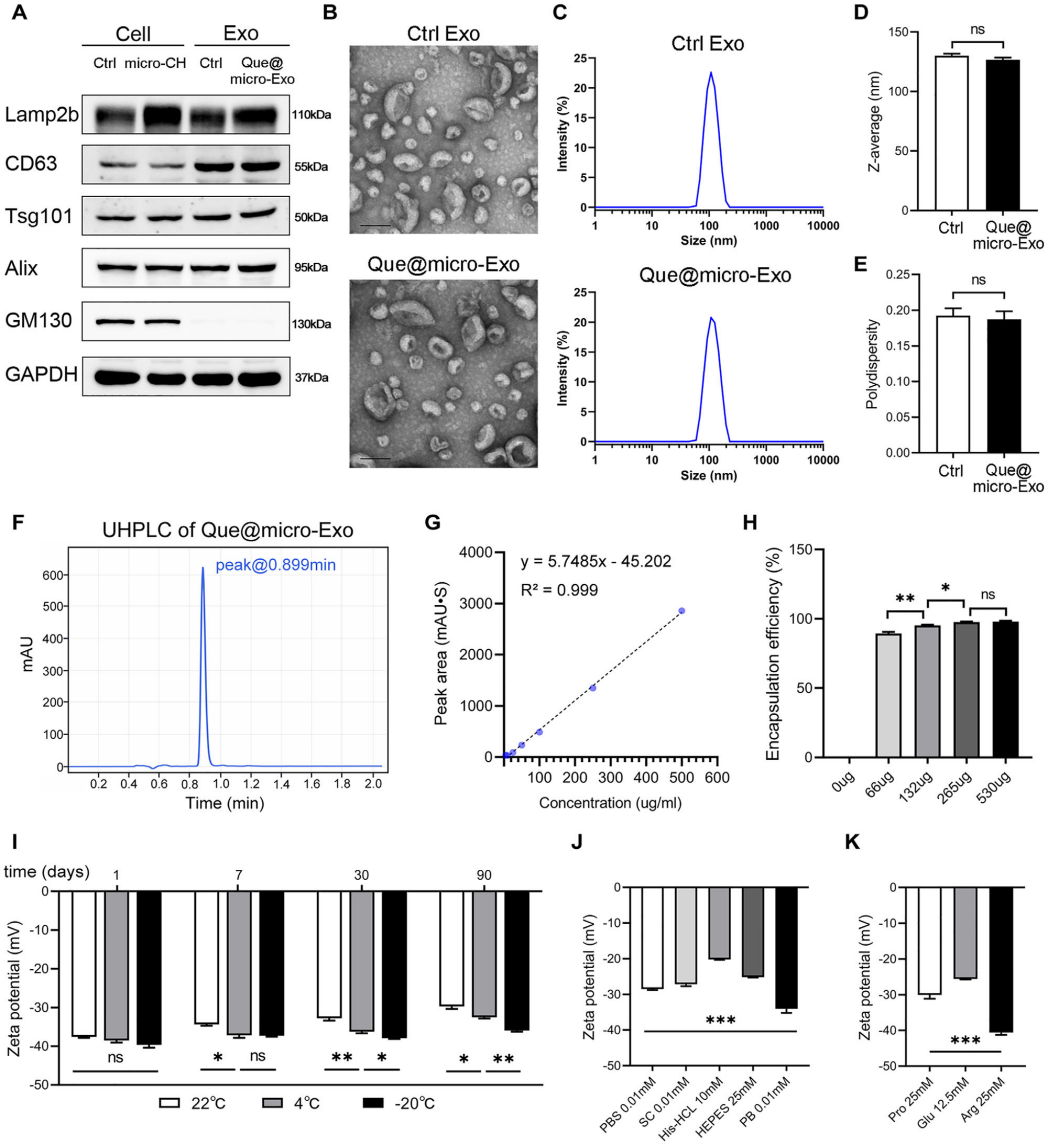
The quercetin‐loaded microglia‐targeted exosomes (Que@micro‐Exo) were constructed and characterized. (A) Western blotting of Lamp2b, CD63, Tsg101, Alix, GM130, and GAPDH in cells transfected with pcDNA3.1(+) plasmid (Ctrl group), pcDNA3.1(+)‐microCH plasmid (micro‐CH group), and in control exosomes (Ctrl Exo) and quercetin‐loaded micro‐exosomes (Que@micro‐Exo). (B) Representative transmission electron microscopy images. Scale bar = 100 nm. (C–E) Size distribution, *Z*‐average size, and Polydispersity index (PDI) of Ctrl Exo and Que@micro‐Exo. *n* = 4–6 per group. (F,G) Ultra‐high performance liquid chromatography (UHPLC) analysis and calibration curve. (H) Encapsulation efficiency of quercetin within the exosomes. (I) Zeta potential of Que@micro‐Exo stored at 22°C, 4°C, and −20°C for 1, 7, 30, and 90 days. (J) Zeta potential of Que@micro‐Exo in different buffers: PBS 0.01 mM, sodium citrate (SC) 0.01 mM, Histidine‐HCl (His‐HCl) 10 mM, HEPES 25 mM, PB 0.01 mM. (K) Zeta potential of Que@micro‐Exo in the presence of 0.01 mM PB with 25 mM proline (Pro), 12.5 mM glutamate (Glu), and 25 mM arginine (Arg). *n* = 3 per group. Data are expressed as mean ± SEM. Statistical analysis was performed using an unpaired two‐tailed Student's *t*‐test and one‐way ANOVA followed by Tukey's post‐hoc test. *p < 0.05; **p < 0.01; ***p < 0.001; ns, not significant.

To produce clinical‐grade exosome products, suspension 293F cells were cultured under feeder‐free conditions in a Good Manufacturing Practices (GMP)‐compliant medium. To generate micro‐CH decorated exosomes (micro‐Exo), recombinant vectors was constructed and transfected into suspension 293F cells. The culture medium underwent sequential ultracentrifugation to isolate micro‐exo, which were subsequently filtered through a 0.22 µm filter to ensure sterilization. The yield per 100 mL of culture medium from five independent batches was 2.71, 1.85, 2.51, 2.68, and 5.36 mg, with an average yield of 3.02 ± 0.61 mg of exosomal proteins per 100 mL. This yield is substantially higher than previously reported values (<0.1 mg per 100 mL) and sufficient for preclinical dosing applications [17]. Leveraging the lipophilicity of quercetin, engineered micro‐Exo were mixed with quercetin to harvest Que@micro‐Exo. We then examined the exosomal markers Lamp2b, CD63, Tsg101, Alix and the Golgi apparatus marker GM130 for both parental cell and exosome samples (Figure 4A). As expected, micro‐CH‐Lamp2b fusion protein was overexpressed in engineered group compared with control group (Ctrl Exo). CD63, Tsg101 and Alix were expressed in both cell and exosomes, while GM130 was hardly detected in exosomes. Representative images of transmission electron microscopy (TEM) showed that the loading of quercetin did not alter the morphology of the exosomes (Figure 4B). Cells and exosomes were all tested negative for mycoplasma. Endotoxin analyses revealed low levels of endotoxins in both Ctrl Exo and Que@micro‐Exo (0.11 ± 0.01 and 0.13 ± 0.01 EU/mL, respectively).

Size distribution and the *Z*‐average size showed that the average size in the control group (130.1 nm ± 1.8 nm) and Que@micro‐Exo (126.7 nm ± 1.9 nm) had no significant difference (Figure 4C,D). The polydispersity index (PDI), which measures the uniformity of particle size distribution, was around 0.19 for both groups (Figure 4E). This PDI value, well below the acceptable threshold of 0.3 and within the ideal range below 0.2, indicates high homogeneity. These results confirm that the engineering process maintains the physical characteristics of the exosomes.

Using Ultra‐High Performance Liquid Chromatography (UHPLC) analysis, we quantified the amount of quercetin in Que@micro‐Exo (Figure 4F). The chromatogram exhibited a distinct peak at 0.899 min, confirming the presence of quercetin within the exosomes. The calibration curve demonstrated a strong linear relationship between peak area and quercetin concentration, with an *R* ^2^ value of 0.999, indicating high accuracy and reliability of the quantification method (Figure 4G). We then examined the encapsulation efficiency and drug‐loading efficiency of quercetin mixed with 200 µg micro‐Exo at varying quercetin concentrations (0, 66, 132, 265, and 530 µg) (Figure 4H and Figure S6). The encapsulation efficiency and drug‐loading efficiency significantly increased with higher quercetin concentrations. We adopted the formula of 200 µg micro‐Exo and 265 µg quercetin (approximately 10.6 mg quercetin/kg) for subsequent experiments.

Zeta potential (ZP) measurement, a critical parameter for assessing the stability of colloidal dispersions, was employed to evaluate the storage stability of Que@micro‐Exo. A zeta potential value of ±30 mV or higher is generally regarded as ideal for ensuring a highly stable colloidal system with minimal aggregation tendencies. Considering the importance of storage stability for off‐the‐shelf drugs, we tested Que@micro‐Exo stored at three different temperatures (22°C, 4°C, and −20°C) over 90 days [18]. These temperatures are commonly achievable in hospital settings. On Day 1, there were no significant differences among the storage conditions (Figure 4I). From Day 7 to Day 90, samples stored at −20°C remained stable, whereas significant zeta potential reduction was observed at 4°C and 22°C. Additionally, we evaluated the *Z*‐average size stability (Figure S7), which revealed that storage at −20°C is the most effective condition for preserving particle size stability. This observation aligns with the findings on zeta potential stability. Overall, these results suggest that storage temperature impacts the stability of Que@micro‐Exo, with −20°C proving to be the optimal condition for maintaining stability over an extended period.

To ensure the stability of Que@micro‐Exo for intravenous application, we examined the effects of different buffers. The figure shows that Que@micro‐Exo in 0.01 mM PBS, 0.01 mM sodium citrate (SC), 10 mM His‐HCL, and 25 mM HEPES exhibited a significant decrease in zeta potential compared to 0.01 mM PB (Figure 4J). Subsequently, we used 0.01 mM PB as the solvent and tested the influence of different pharmaceutical excipients. The results indicated that, compared with 25 mM proline (Pro) and 12.5 mM glutamate (Glu), 25 mM arginine (Arg) provided significantly better stability (Figure 4K). This indicates that a formulation consisting of 0.01 mM PB and 25 mM arginine is an optimal buffer system for Que@micro‐Exo agent, ensuring its stability as an intravenous agent.

### 2.5 Modified Exosomes Effectively Target CD86‐Positive Microglia in the Ischemic Region

**FIGURE 5:**
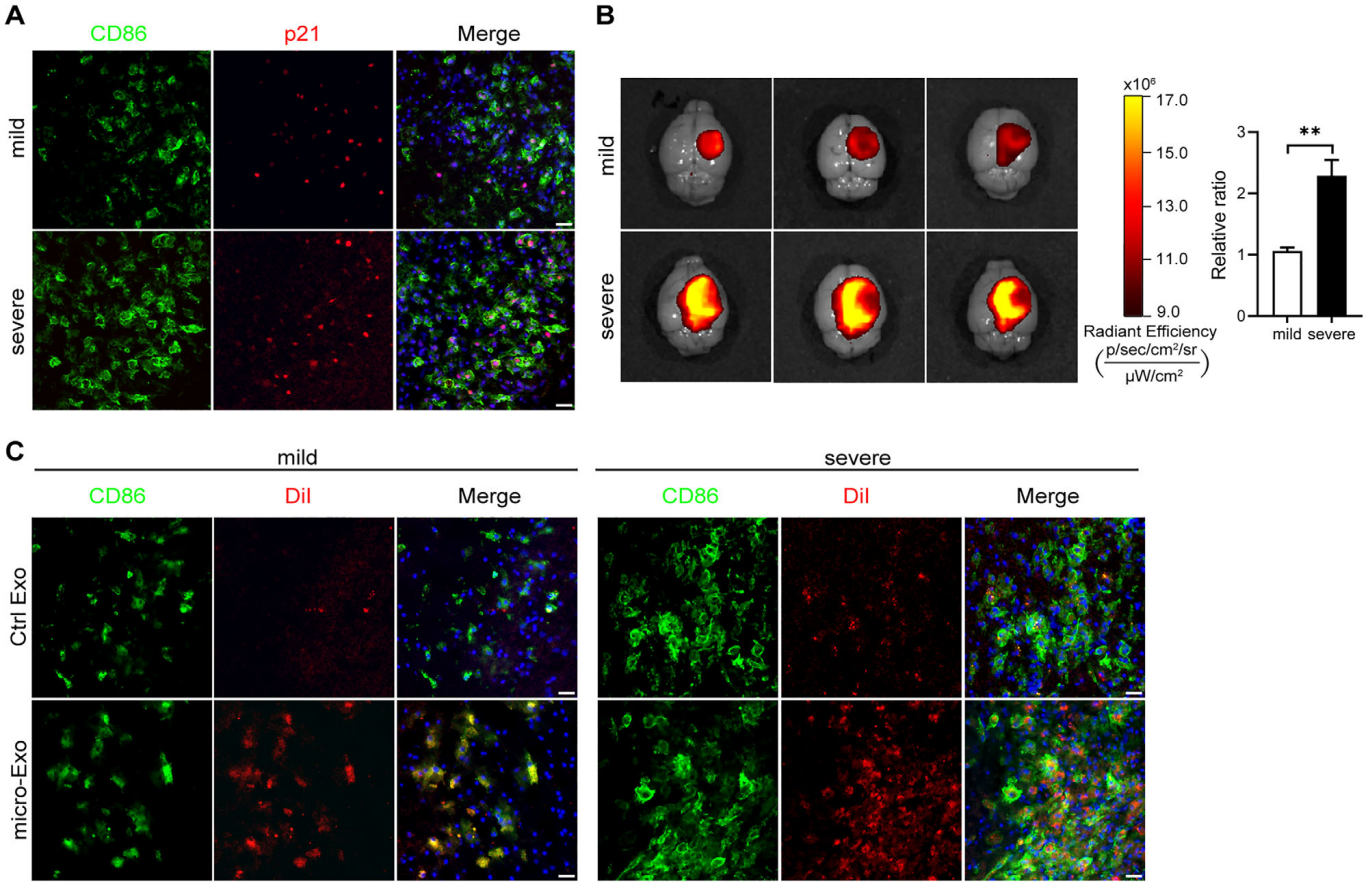
Modified exosomes effectively target CD86‐positive microglia in the ischemic region. (A) Representative immunofluorescence images of CD86 and p21 in mild and severe ischemic models at 3dpi. (B) Representative in vivo IVIS tracking images and quantitative analysis showing the distribution of DiR‐labeled micro‐Exo. (C) Representative immunofluorescence images of CD86 and DiI in mild and severe ischemic models at 3 dpi. Scale bar = 30 µm. Data are expressed as mean ± SEM, with *n* = 3 per group. Statistical analysis was performed using an unpaired two‐tailed Student's *t*‐test. **p < 0.01.

To investigate the targeting ability of micro‐CH‐decorated exosomes (micro‐Exo) in CD86‐positive microglia within ischemic regions, we established mild and severe ischemic models. Immunofluorescence analysis at 3 dpi revealed a higher number of p21^+^CD86^+^ microglia in the severe ischemic model compared to the mild model, indicating an increased inflammatory phenotype (Figure 5A). To visualize the distribution of micro‐Exo in ischemic region, we labeled 200 µg micro‐Exo with DiR dye and administered it via tail vein injection. Mice were sacrificed 4 h post‐injection for in vivo IVIS tracking (Figure 5B). Representative IVIS images showed that severe ischemic condition exhibited higher signals in the ischemic regions, as indicated by the more intense yellow–red color. Quantitative analysis confirmed significantly increased accumulation of micro‐Exo in the ischemic region in the severe model.

To further validate these findings, we labeled 200 µg micro‐Exo or 200 µg Ctrl Exo with DiI dye and injected them into the tail vein 4 h post‐ischemia (Figure 5C). Mice were sacrificed at 3 dpi. In the Ctrl Exo group, a small number of dot‐shaped DiI signals were observed at the site of cerebral ischemic injury. These signals increased modestly as the degree of cerebral ischemia worsens, but notably, they lacked the typical cellular morphology associated with successful targeting (Figure 5C, upper panel). In contrast, the micro‐Exo group showed a significant increase in DiI and CD86 double‐positive cells (Figure 5C, lower panel). As the severity of injury increased, more DiI and CD86 double‐positive cells were detected, consistent with the IVIS observations. The co‐localization of DiI and CD86 signals confirmed the successful targeting and internalization of micro‐Exo by CD86‐positive microglia. Additionally, DiI signals were observed in the reticuloendothelial system, such as the liver, spleen, and lung, while few in heart and kidney (Figure S8). Notably, slightly weaker fluorescent signals were seen in these organs in the micro‐Exo group. These results indicate that micro‐CH modification of micro‐Exo enhances targeting to CD86‐positive microglia in ischemic regions, potentially reducing off‐target accumulation in the reticuloendothelial system. These novel findings suggest that micro‐Exo can effectively target and accumulate in CD86‐positive microglia in ischemic regions, offering a promising strategy for enhancing the therapeutic efficacy of exosome‐based senolytic delivery treatments for ischemic stroke.

### 2.6 Que@micro‐Exo Mitigates Ischemic Injury and Enhances Recovery

**FIGURE 6:**
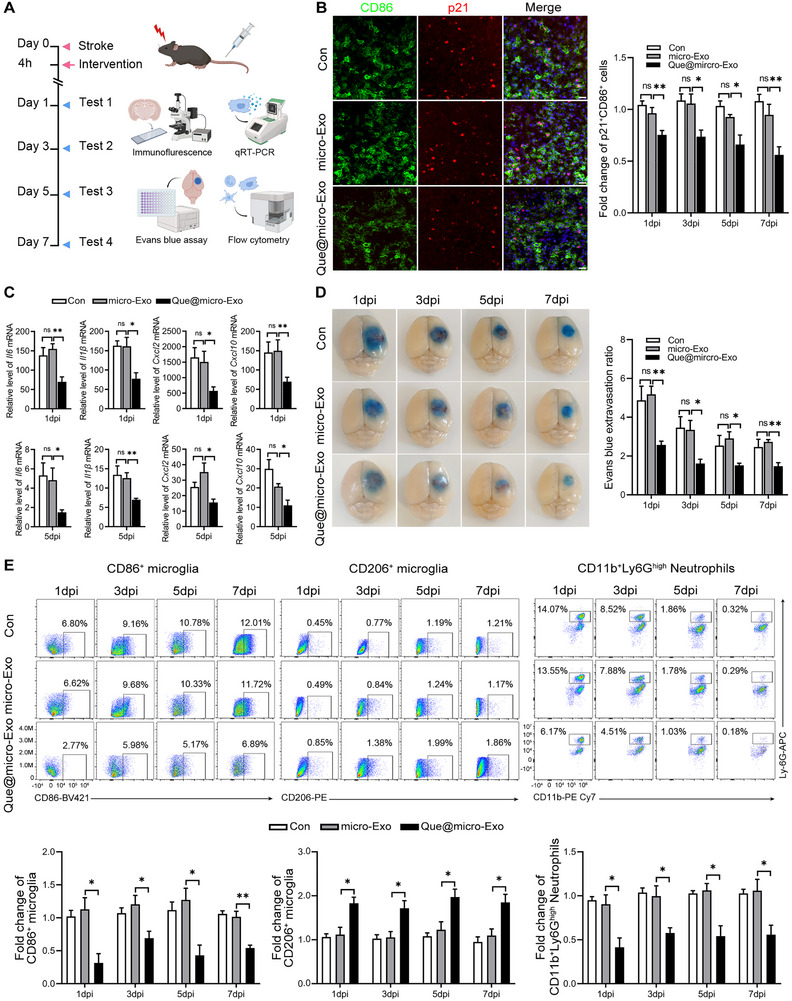
Que@micro‐Exo therapy mitigates cerebral ischemic injury. (A) Schematic diagram outlining the experimental timeline for stroke induction, Que@micro‐Exo intervention, and subsequent assessments to evaluate the therapeutic effects between the control group (Con), microglia‐targeted exosomes (micro‐Exo) group and quercetin‐loaded micro‐exosomes (Que@micro‐Exo) group. (B) Representative immunofluorescence images and quantitative analysis of CD86/p21 at 7dpi. Scale bar = 30 µm. *n* = 3 per group. (C) qRT‐PCR analysis showing the expression levels of *Il6*, *Il1β*, *Cxcl2*, and *Cxcl10* in the ischemic region at 1 and 5dpi. *n* = 4 per group. (D) Representative brain images of Evans blue assay and quantitative analysis at 1, 3, 5, and 7dpi. *n* = 3 per group. (E) Representative flow cytometry plots and quantitative analysis of CD86^+^ M1 microglia, CD206^+^ M2 microglia, and CD11b^+^Ly6G^high^ neutrophils in the ischemic region at 1, 3, 5, and 7dpi. *n* = 3 per group. Data are expressed as mean ± SEM. Data were analyzed by an unpaired two‐tailed Student's *t*‐test. *p < 0.05; **p < 0.01; ***p < 0.001; ns, not significant.

To test the efficacy of Que@micro‐Exo agent, we injected 200 µL control buffer (consisted of 0.01 mM PB and 25 mM arginine), 200 µg micro‐Exo in 200 µL control buffer, and 200 µL Que@micro‐Exo agent into the tail vein only once at 4 h post ischemia (Figure 6A). Immunofluorescence analysis revealed a significant reduction in the number of p21^+^CD86^+^ microglia in the Que@micro‐Exo group compared to controls (Figure 6B). To further elucidate the anti‐inflammatory effects of Que@micro‐Exo, we measured the expression of *Il6*, *Il1β*, *Cxcl2*, and *Cxcl10* at 1 and 5 dpi using qRT‐PCR (Figure 6C). The results demonstrated a significant decrease in these pro‐inflammatory cytokines following Que@micro‐Exo treatment.

Previous studies have highlighted the detrimental roles of *Il6*, *Il1β*, *Cxcl2*, and *Cxcl10* in promoting BBB breakdown, microglial polarization, and neutrophil infiltration. Thus, we examined these pathological processes in our model. We assessed BBB integrity using Evans blue extravasation at 1, 3, 5, and 7 dpi (Figure 6D). In the control group, extensive blue staining in the ischemic regions at 1 dpi indicated significant BBB breakdown. This staining persisted at 3, 5, and 7 dpi, with only slight reductions over time. Similar patterns were observed in the micro‐Exo group. However, the Que@micro‐Exo group showed significant Evans blue extravasation at 1 dpi, but from 3 dpi onward, a noticeable reduction was observed compared to both the control and micro‐Exo groups. By 5 and 7 dpi, the Que@micro‐Exo group exhibited markedly reduced Evans blue extravasation, suggesting improved BBB integrity and reduced breakdown over time. This indicates that Que@micro‐Exo mitigates BBB damage in cerebral ischemia. Microglial polarization and neutrophil infiltration were assessed via flow cytometry at 1, 3, 5, and 7 dpi (Figure 6E). The cell sorting strategy is detailed in the supplementary figure (Figure S9). Flow cytometry plots indicated a significant decrease in CD86‐positive M1 microglia and neutrophils, alongside a significant increase in CD206‐positive M2 microglia in the Que@micro‐Exo group. These findings collectively suggest that Que@micro‐Exo exerts a protective effect in cerebral ischemia by reducing inflammation, enhancing BBB integrity, and promoting beneficial microglial polarization.

**FIGURE 7:**
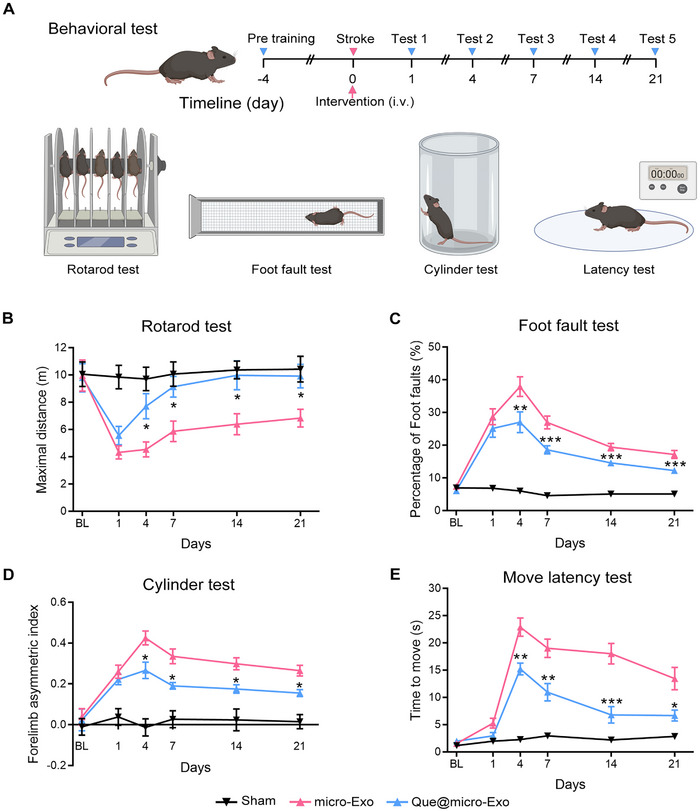
Que@micro‐Exo therapy improves functional recovery after cerebral ischemia. (A) Schematic diagram outlining the experimental timeline for pre‐training of behavioral tests, stroke induction, Que@micro‐Exo intervention, and subsequent assessments of functional recovery among the sham group (Sham), microglia‐targeted exosomes (micro‐Exo) group and quercetin‐loaded micro‐exosomes (Que@micro‐Exo) group. (B–E) Results of rotarod test, foot fault test, cylinder test, and move latency test. Data are expressed as mean ± SEM, with *n* = 6 per group. Data were analyzed by an unpaired two‐tailed Student's *t*‐test. *p < 0.05; **p < 0.01; ***p < 0.001.

Next, we tested whether Que@micro‐Exo therapy could promote functional recovery. We pretrained mice for the rotarod test, foot fault test, cylinder test, and move latency test for 4 days, and then examined the functional performance at 1, 4, 7, 14, and 21 dpi (Figure 7A). The results showed that the Que@micro‐Exo group exhibited significant recovery starting from day 4, with marked improvements continuing through days 7, 14, and 21 (Figure 7B–E). As measured by indicators such as maximal distance traveled on the rotarod, percentage of foot faults, forelimb asymmetry index, and time to move, the performance in the Que@micro‐Exo group significantly improved compared to both sham‐operated mice and those receiving micro‐Exo treatments.

### 2.7 Safety Evaluation of Que@micro‐Exo Therapy

**FIGURE 8:**
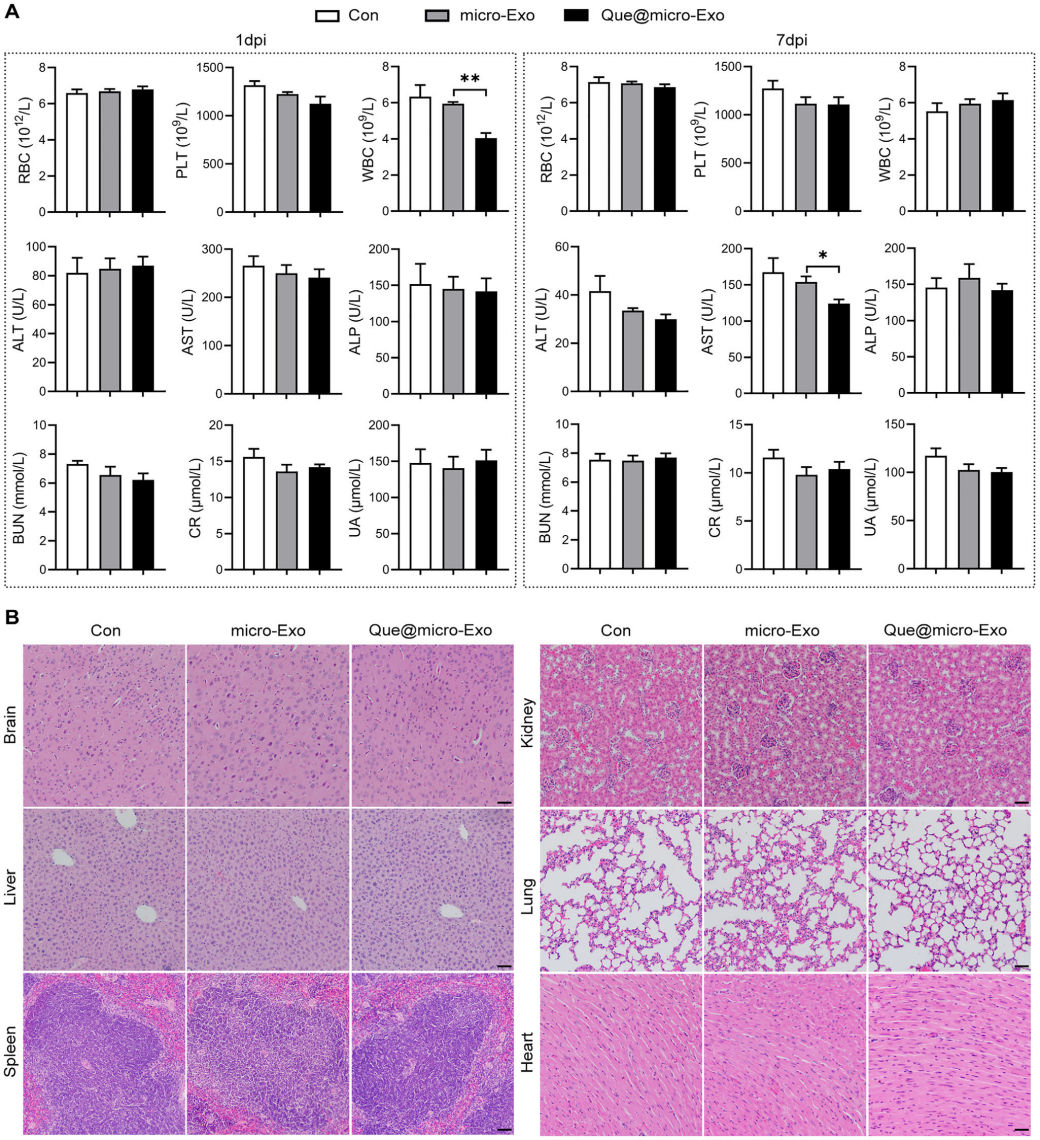
Que@micro‐Exo treatment for cerebral ischemia in mice was found to be safe. (A) Hematological and biochemical analyses evaluating the safety among the control group (Con), microglia‐targeted exosomes (micro‐Exo) group, and Quercetin‐loaded micro‐exosomes (Que@micro‐Exo) group. Parameters include red blood cells (RBC), platelets (PLT), white blood cells (WBC), alanine aminotransferase (ALT), aspartate aminotransferase (AST), alkaline phosphatase (ALP), blood urea nitrogen (BUN), creatinine (CR), and uric acid (UA) at 1 and 7 dpi. (B) Hematoxylin and eosin (H&E)‐stained pathological sections of major organs (brain, liver, spleen, kidney, lung, and heart) at 21 dpi. Scale bar = 40 µm. Data are expressed as mean ± SEM, with *n* = 5 per group. Data were analyzed by an unpaired two‐tailed Student's *t*‐test. *p < 0.05; **p < 0.01.

To evaluate the safety of Que@micro‐Exo therapy, we performed comprehensive hematological and biochemical analyses. Routine indexes including red blood cells (RBC), platelets (PLT), white blood cells (WBC), alanine aminotransferase (ALT), aspartate aminotransferase (AST), alkaline phosphatase (ALP), blood urea nitrogen (BUN), creatinine (CR), and uric acid (UA) were examined at 1 and 7 dpi (Figure 8A).

No significant changes were observed in RBC, PLT, ALP, BUN, CR, and UA levels across all groups at both time points, and all data fell within the normal range. At 1 dpi, the Que@micro‐Exo group showed a significant reduction in WBC counts compared to the control and micro‐Exo groups. However, by 7 dpi, WBC counts had normalized, with no significant differences among the groups. In terms of liver function, we noted that ALT and AST levels, which typically range from 20 to 80 U L^−1^ and 50 to 150 U L^−1^, respectively, in 9‐week‐old mice, were elevated across all groups at 1 dpi without significant intergroup differences. Subsequent testing of ischemic mice injected with saline showed similar ALT and AST levels (87.40 ± 7.00 U L^−1^ and 262.60 ± 15.17 U L^−1^, respectively), indicating that the elevated enzyme levels were likely due to the ischemic model induction. By 7 dpi, the Que@micro‐Exo group exhibited a significant reduction in AST levels to 124.20 ± 5.48 U L^−1^, remaining within the normal range, compared to the control groups. This finding suggests that non‐specific uptake of Que@micro‐Exo by the liver may help mitigate liver aminotransferase abnormalities, suggesting a potential hepatoprotective effect of Que@micro‐Exo therapy.

To assess the impact of Que@micro‐Exo treatment on tissue structure, we prepared H&E‐stained pathological sections of major organs (brain, liver, spleen, kidney, lung, and heart) at 24 h, 7 and 21 dpi (Figure 8B). Visual observation and pathological analysis revealed no significant abnormalities such as tissue disorganization, immune cell infiltration, or fibrosis in the examined organs.

## 3 DISCUSSION

In this study, we identified the aberrant accumulation of p21^+^CD86^+^ microglia in the ischemic regions of both mice and humans, characterizing their dominant proinflammatory phenotype. Building on this discovery, we developed an exosome‐based senolytic therapy, Que@micro‐Exo, which effectively targets and eliminates p21^+^CD86^+^ microglia post‐ischemia. This intervention significantly reduced cerebral ischemic injury and enhanced functional recovery with safety. Our findings underscore the clinical potential of Que@micro‐Exo in treating stroke and other cellular senescence‐associated conditions, including age‐related diseases, by offering a novel strategy to mitigate inflammation and cellular senescence in ischemic injury.

Cellular senescence has been associated with neurological diseases, and the genetic or pharmacological ablation of the senescent response has shown benefits. Recent studies highlighted the importance of investigating senescence heterogeneity in cell types and tissues. In the context of aging and neurodegenerative disease, senescent cell accumulation has been identified in various CNS cell types, including neurons, microglia, astrocytes, oligodendrocyte precursor cells, and endothelial cells [19]. However, the role of cellular senescence and SASP factors in ischemic brain injury remains poorly described.

Stroke induces hypoxia, neuroinflammation, oxidative stress, DNA damage, and mitochondrial dysfunction, all contributing to the induction of senescence. Microglia, essential for CNS homeostasis through environmental surveillance and phagocytic clearance, are key players in neuroinflammatory cascades during stroke. Previous studies have observed elevated levels of senescence markers such as p21 and p16, along with various SASP factors, in infarct regions [20, 21]. Our research identified p21 as a more dominant marker than p16 in microglia post‐ischemia, particularly in CD86‐positive M1 microglia. Single‐cell analysis revealed that these p21^+^CD86^+^ microglia exhibit a prominent pro‐inflammatory phenotype, upregulating SASP components such as *Il6*, *Il1β*, *Cxcl2*, and *Cxcl10*. Several intracellular pathways have been reported to regulate SASP expression, including mTOR, p38 MAPK, JAK/STAT, and the inflammasome, leading to the activation of transcription factors such as NF‐kB and C/EBP [22]. The specific mechanisms of SASP activation in ischemic stroke, however, remain unexplored. Clinical meta‐analyses and cohort datasets have demonstrated that elevated levels of *Il6*, *Il1β*, *Cxcl2*, and *Cxcl10* are closely associated with poorer stroke prognosis. Basic research indicates these cytokines exacerbate immune cell infiltration, increase BBB breakdown, and perpetuate the production of more harmful chemokines and cytokines [23, 24, 25, 26]. Through bioinformatics prediction [27, 28, 29, 30], CoIP, ChIP, EMSA, and dual‐luciferase assays, we revealed a novel p21‐C/EBPβ interaction, confirming C/EBPβ‐regulated SASP release. These findings lay the foundation for targeting p21^+^CD86^+^ microglia to improve stroke prognosis, providing compelling mechanism‐based evidence for the clinical potential of our exosome‐based senolytic therapy.

Targeting senescent cells that accumulate in disease‐related tissues presents a promising strategy to alleviate disease burden. Currently, preclinical studies and clinical trials focus primarily on broad and non‐specific senolytic therapies, such as dasatinib plus quercetin, fisetin, inhibitors of antiapoptotic BCL‐2 family proteins (e.g., navitoclax/ABT‐263, ABT‐737), p53 modulators like UBX0101, and metformin [22, 31]. These approaches lack specificity, failing to distinguish between different cell types or senescence markers. Ideally, a targeted therapy would selectively eliminate harmful senescent cells while sparing non‐pathogenic ones. Given the heterogeneity of senescent cells, the prerequisite for cell‐type‐specific therapy is identifying the target subpopulation. In the context of ischemic stroke, we identified detrimental p21^+^CD86^+^ microglia in the infarct region, providing a novel therapeutic target. Addressing the challenge of targeted drug delivery, exosome‐based approaches have garnered considerable attention. Exosomes, as endogenous information carriers, offer advantages such as the ability to cross the blood‐brain barrier, low immunogenicity, and potential for targeted modification [32]. Compared to viral vectors, liposomes, nanoparticles, and polymer micelles, modified exosomes provide a superior platform for the delivery of biologically active drugs in neurological diseases. Our study developed Que@micro‐Exo by fusing CD86‐positive microglia targeting peptide with exosomal protein Lamp2b to efficiently deliver quercetin. Leveraging single‐cell analysis to identify more cell markers for heterogeneous senescent cells in future studies, there is potential to develop therapeutic exosome cocktails by decorating them with peptides targeting various cell markers.

To date, the most well‐studied senolytic cocktail is dasatinib plus quercetin, with over eight clinical trials completed, ongoing, or planned, targeting conditions such as Alzheimer's disease, diabetic kidney disease, and idiopathic pulmonary fibrosis [22, 31, 33]. In our study, we focused on exosome‐delivered quercetin monotherapy. Quercetin has proven effective in eliminating senescent cells and exhibits anti‐inflammatory, antioxidant, and antineoplastic properties, but suffers from low solubility, instability, and bioavailability [34, 35]. Dasatinib, approved for leukemia, is associated with frequent adverse events typical of antitumor drugs, including myelosuppression, headaches, and fluid retention, with incidences often exceeding 20% [36]. Critically, dasatinib may induce platelet dysfunction. The European Union Summary of Product Characteristics (SmPCs) advises caution when administered with antiplatelet agents or anticoagulants, which are recommended for ischemic stroke patients by the American Heart Association, barring contraindications. Recent computational studies have identified non‐toxic analogs of dasatinib, particularly from natural sources, that could synergistically enhance quercetin's senolytic efficacy while minimizing adverse effects [37]. These alternatives merit further investigation for their potential to provide a safer and equally effective therapeutic approach.

Recent years have seen a significant increase in clinical trials assessing the safety, tolerability, and preliminary efficacy of exosome‐based therapies. Over 70 clinical trials involving therapeutic exosomes for various indications are registered on ClinicalTrials.gov and the Chinese Clinical Trial Registry [38]. Notably, Aruna Bio's investigational new drug (IND) application for AB126, naïve exosomes derived from neural stem cells, has received FDA clearance, marking the first exosome to advance to a Phase 1b/2a clinical trial for acute ischemic stroke. However, the low yield of exosome production remains a significant limitation, particularly for naïve exosomes. Compared with naïve exosomes, modified exosomes are primarily derived from HEK293 cells, which have been commonly used in research and the bio‐industrial field for over 30 years. Our research group has previously utilized mesenchymal stem cells and adherent HEK293 cells for exosome manipulation [9, 10]. In this study, we employ suspension 293F cells, which offer notable advantages for GMP‐compliant, safety with minimal toxicity and immunogenicity, ease of genetic modification, scalability for bioreactors, and large‐scale production of industrial bioproducts. HEK293‐derived biopharmaceuticals have recently received approvals from the FDA and the European Medicines Agency (EMA), underscoring their potential for clinical‐grade therapeutic exosome production [39].

We incorporated quercetin into exosomes through simple physical co‐incubation, a method chosen for its simplicity, scalability, and compatibility with the lipophilic nature of quercetin. This approach facilitates the establishment of a scalable biomanufacturing workflow to produce clinical‐grade Que@micro‐Exo for future preclinical and clinical applications. Our study achieved an encapsulation efficiency of approximately 98.06% and a drug‐loading efficiency of around 55%, demonstrating both the feasibility and therapeutic potential of this method. While co‐incubation proved effective in achieving high encapsulation and drug‐loading efficiencies, further optimization of loading conditions, such as adjusting temperature, pH, and quercetin‐to‐exosome ratios, could further enhance the efficiency of this system. Additionally, alternative loading strategies, such as surface modifications or electroporation, may warrant investigation for specific applications, though their scalability remains a challenge. The optimization of Que@micro‐Exo additives has demonstrated high stability, favorable zeta potential, and achievable storage conditions, positioning Que@micro‐Exo as a promising off‐the‐shelf therapeutic agent. Future investigations should explore lyophilization techniques to enable room‐temperature storage, further enhancing preservation and transport convenience for clinical applications.

Despite promising outcomes in murine ischemic models, Que@mico‐Exo therapy's efficacy in non‐human primates or humans remains untested. Que@micro‐Exo is designed to identify and eradicate p21^+^CD86^+^ microglia. CD86 is a conserved protein across mammalian species, with a high degree of sequence homology and cross‐species functionality, supported by experimental evidence in mice and humans [40, 41]. Thus, it is worth to translate Que@mico‐Exo therapy from bench to bedside for clinical assessment. However, CD86 is rapidly upregulated on macrophages, B cells, dendritic cells, and on other antigen‐presenting cells in response to inflammation or infections [42, 43]. Consequently, Que@mico‐Exo may inadvertently target these cells, risking tissue damage and adverse effects. Therefore, a comprehensive evaluation of these risks is essential when considering the use of Que@mico‐Exo therapy in individuals with inflammatory and infectious diseases.

Additionally, our study identified a potential hepatoprotective effect of Que@micro‐Exo, which may be attributed to non‐specific uptake in the liver. The liver‐protective effects of quercetin are well‐documented and are likely mediated by multiple mechanisms, including its anti‐inflammatory effects via the NF‐κB/TLR/NLRP3 pathways, antioxidant activity through the PI3K/Nrf2 pathway, antifibrotic effects via the TGF‐β/Smad pathway, and lipid metabolism regulation through the p62/mTOR/CD36 axis [44]. Clinical trials have also confirmed quercetin's hepatoprotective properties, demonstrating its ability to reduce liver injury markers and improve liver function [45]. These findings collectively suggest that Que@micro‐Exo may exert hepatoprotective effects, warranting further investigation to fully elucidate the underlying mechanisms and optimize its therapeutic potential.

Current guidelines by the American Heart Association/American Stroke Association recommend early thrombolysis within 4.5 h of symptom onset for acute ischemic stroke management. We administered a single dose of Que@mico‐Exo therapy at 4 h post‐ischemia, which theoretically does not conflict with thrombolytic drugs. Moreover, this application may be synergistic with thrombolytic drugs and potentially play a neuroprotective role in enhancing stroke recovery. Considering the hit‐and‐run mechanism of senolytics, intermittent administration, such as weekly dosing, may be crucial in both acute and chronic phases of stroke. Therefore, optimizing intravenous dosages and delivery schedules of Que@mico‐Exo therapy is essential for maximizing its therapeutic efficacy.

## 4 MATERIALS AND METHODS

### 4.1 Animals and Photothrombotic Model

Male C56BL/6 mice, aged 8–9 weeks and weighing 24–25 g, were used in this study. Ethical approval for all animal experiments was obtained from the Animal Care and Use Committee of the Sino Animal (Beijing) Science and Technology Development Co., Ltd (20230127YZA‐3R). Focal cortical ischemia was made as previously described. Briefly, mice received a tail‐vein injection of Rose Bengal at a dosage of 25 mg kg^−1^. The skull window ranging from 0.5 to 2.0 mm posterior to the bregma and 0.0 to 2.0 mm right to the midline. For mild and severe ischemic model establishment, the skull window was illuminated for 5 or 8 min by an optic fiber connected to a 532‐nm light source. The sham group mice were subjected to identical procedures, with the exception of light illumination. To investigate the effects of exosomes in vivo, 200 µg Ctrl Exo, 200 µg micro‐Exo, and Que@micro‐Exo agent were injected into the tail vein only once at 4 h post ischemia. All studies adhered to the ARRIVE (Animal Research: Reporting of In Vivo Experiments) guidelines. Mice were randomized into different groups, and sample sizes were determined based on pilot studies and previous experimental experience to ensure adequate statistical power in line with field standards. The number of biological replicates for each experiment is detailed in the figure legends. The analysis involving animal studies was conducted in a double‐blinded fashion, with no data exclusions.

### 4.2 Cell Culture

The BV2 murine microglial cell lines and human embryonic kidney (HEK) 293 cells were purchased from the National Infrastructure of Cell Line Resource. BV2 and HEK 293 Cells were cultured in high glucose Dulbecco's modified Eagle's medium (DMEM) (Thermo Fisher Scientific), supplemented with 10% fetal bovine serum (FBS) (Thermo Fisher Scientific) and 1% penicillin/streptomycin (P/S) (Thermo Fisher Scientific). The cells were maintained at 37°C in a humidified atmosphere containing 5% CO_2_ and passaged every 2 to 3 days. Suspension Expi293F (293F) cells were purchased from Thermo Fisher Scientific and cultured in Expi293 Expression Medium. These cells were maintained in Nalgene single‐use Erlenmeyer flasks on an orbital shaker (Infors, Multitron) with a 25 mm shaking diameter at a speed of 120 rpm. The incubation conditions were set at 37°C with 80% humidity and 8% CO₂.

### 4.3 Exosome Engineering and Characterization

The sequence expressing homing peptide CHHSSSARC (micro‐CH) fused with exosomal protein Lamp2b was cloned into pcDNA3.1(+) vector by GenScript Biotech Corporation. To produce Ctrl Exo and micro‐Exo, 293F cells were transfected with pcDNA3.1(+) and pcDNA3.1(+)‐microCH plasmids by using ExpiFectamine 293 Reagent (Thermo Fisher Scientific). 293F cells were cultured for 72 h and exosomes were isolated through sequential ultracentrifugation. Harvested culture medium was centrifuged at 300 × *g* for 5 min to remove cells, at 2000 × *g* for 10 min to remove cell debris, and underwent ultracentrifugation at 100,000 × *g* using the Avanti JXN‐30 (Beckman) at 4°C for 2 h. To ensure sterilization, the resuspended exosomes were filtered with 0.22 µm filter. To produce Que@micro‐Exo, quercetin (Solarbio) of different doses was mixed with 200 µg isolated micro‐Exo in 1 mL PBS at 37°C for 1 h by using ThermoMixer C (Eppendorf AG) and centrifugated at 14,000 g for 1 h to pellet the Quercetin‐loaded micro‐exosomes (Que@micro‐Exo). The resulting pellet was washed twice with PBS, with each wash followed by centrifugation at 14,000 g for 1 h, to remove any unencapsulated quercetin. The exosomal protein was measured by Pierce BCA Protein Assay Kit (Thermo Fisher Scientific). The size distribution of exosomes, presented as *Z*‐average and polydispersity index (PDI), along with the stability of exosomes indicated by zeta potential, were determined using dynamic light scattering with the Zetasizer Ultra instrument (Malvern Panalytical Ltd). Measurements were based on an intensity‐based algorithm, and data processing and analysis were conducted using the ZS Xplorer software (Malvern Panalytical Ltd). For DiI in vivo fluorescence labeling and transmission electron microscopy, the protocol was carried out as previously described by our group (8).

### 4.4 Ultra High‐Performance Liquid Chromatography (UHPLC) Analysis

Quercetin detection kit (Solarbio) was used for the UHPLC analysis of quercetin. A series of quercetin standard solutions with known concentrations were prepared and a calibration curve (*y*‐axis: absorbance in milli‐Absorbance Units (mAU); *x*‐axis: concentration in µg mL^−1^) was generated according to the manufacture's instruction. Quercetin from Que@micro‐Exo was extracted using the kit's extraction solution. The analysis was performed on an Agilent 1290 Infinity II UHPLC system (Agilent Technologies) equipped with an ACQUITY UPLC HSS‐C18 chromatographic column (2.1 × 100 mm, 1.8 µm) (Waters). The mobile phase consisted of 0.4% phosphoric acid in water (phase A) and acetonitrile (phase B), with a flow rate of 0.5 mL min^−1^. The column chamber temperature was set to 30°C, and UV detection was at a wavelength of 360 nm. Encapsulation efficiency (EE%) is calculated by (total quercetin added—free non‐entrapped quercetin) divided by the total quercetin added.

### 4.5 In Vivo Imaging System (IVIS) Spectrum for Fluorescence Imaging

Exosomes were labeled with DiR (Thermo Fisher Scientific). DiR‐labelled exosomes (200 µg) were intravenously administered into mice via tail vein. Fluorescence imaging of brain was conducted at 4 h interval post‐injection using a small‐animal fluorescence imaging system (Xenogen IVIS Spectrum, Perkin Elmer) with excitation and emission wavelengths set at 745 and 800 nm, respectively. Regions of interest (ROI) were delineated and average radiant efficiency was quantified in units of (p/s/cm^2^/sr)/(µW cm^−2^).

### 4.6 Endotoxin and Mycoplasma Assessment

Exosomes were analyzed by using Pierce Chromogenic Endotoxin Quant Kit (Thermo Fisher Scientific) based on the amoebocyte lysate method. Standard curve was plotted according to the instruction, with the absorbance at OD 405 nm on the *y*‐axis versus the corresponding endotoxin concentration in EU/mL on the *x*‐axis. The endotoxin concentration of each sample was determined by the formulated standard curve (linear regression).

Mycoplasma contamination was assessed using the MycoAlert PLUS Mycoplasma Detection Kit (Lonza). Both the cells and exosomes were lysed using the lysis buffer provided in the kit. The lysates were then reacted with the MycoAlert PLUS Substrate. The luminescence signals were measured using a MULTISKAN FC microplate reader (Thermo Fisher Scientific). A signal ratio of less than 1 indicates the absence of mycoplasma contamination.

### 4.7 Oxygen‐Glucose Deprivation

BV2 cells were transferred to DMEM without glucose (Thermo Fisher Scientific) and subjected to a hypoxic environment using a hypoxia chamber (Billups‐Rothenberg). The chamber containing the cultures was infused with gas mixture of 95% N2 and 5% CO2, and maintained in an incubator with 5% CO2 at 37 C for 3 h to induce oxygen‐glucose deprivation (OGD) injury. Cells maintained in DMEM with glucose under normoxic conditions served as the control group.

### 4.8 Quantitative Reverse Transcription Polymerase Chain Reaction (qRT‐PCR)

Total RNA from cells and tissues was extracted using the TRIzol extraction method. cDNA was synthesized using the PrimeScript RT Master Mix (Takara Bio Inc). qRT‐PCR was performed using TB Green Premix Ex Taq II (Takara Bio Inc). qRT‐PCR primers were designed using the NCBI Primer‐Blast and detailed in Table S1. Relative gene expression was normalized to the reference gene Actb using the 2^−ΔΔ^ *^Ct^* method based on the threshold cycle (*C_t_*) value.

### 4.9 RNA Interference and Bulk RNA Sequencing

The expression of *Cdkn1a* and *C/EBPβ* was inhibited in BV2 cells using small interfering RNAs (siRNAs). The siRNAs and negative control sequences were listed in Table S1. Transfections were conducted using Lipofectamin RNAiMAX (ThermoFisher Scientific) following the manufacturer's protocols. After 48 h of incubation, the siRNA‐transfected cells were used for further experiments. For bulk RNA sequencing, total RNA was extracted from OGD BV2 cells transfected with siRNA negative control and siRNA *Cdkn1a* using TRIzol method. RNA integrity was evaluated with the RNA Nano 6000 Assay Kit of the Bioanalyzer 2100 system (Agilent Technologies). Libraries were sequenced on Illumina NovaSeq 6000 platform (Novogene Co., Ltd.).

### 4.10 Flow Cytometry

The brain tissue of C57BL/6 mice were enzymatically digested by trypsin. To isolate single cells, the samples were filtered twice through a 70 µm cell strainer and washed with 10 mM PBS. For density gradient centrifugation, the pellet was resuspended in 37% Percoll (Cytiva) in 10 mM PBS, underlaid with 70% Percoll, and overlaid with 30% Percoll. The samples were centrifuged at 300 g for 40 min without breaks. The cell layer was then transferred and washed twice with 10 mM PBS. After counting and aliquoting, the cells were stained with Fixable Viability Stain 700 (FVS700) (Becton Dickinson and Co.) to label non‐viable cells. Specific antibodies were used to identify various cell populations, as listed in Table S2: CD45^+^ for immune cells, CD11b^+^Ly6G^high^ for neutrophils, CD11b^+^F4/80^+^Ly6C^−^CD86^+^ for M1 microglia, and CD11b^+^F4/80^+^Ly6C^−^CD206^+^ for M2 microglia. To measure M2 microglia, the cell suspension was pretreated with Cytofix/Cytoperm Fixation/Permeabilization Kit (BD Biosciences). All cells were analyzed using flow cytometer NL‐CLC3000 (Cytek Biosciences) equipped with Cytek's SpectroFlo software. The final data were analyzed using FlowJo software (BD Biosciences).

### 4.11 Single Cell RNA Sequencing Analysis

The ischemic brain tissue was dissociated with the gentleMACS Dissociator (Miltenyi Biotec). The single cell suspension was subjected to 3' single cell RNA sequencing by using 10× Genomics Chromium Single Cell 3' Reagent Kits, and sequencing reads were mapped to the GRCm38 (mm10) reference genome. The GSE number of the dataset at Gene Expression Omnibus (GEO) database was GSE197731. Data analysis was conducted using Seurat version 4.4.0, with filtering criteria including genes detected in more than three cells, cells containing 200 to 6000 genes, unique molecular identifiers from 1000 to 30,000, and mitochondrial gene content below 30%. Additionally, CD45‐negative cells were filtered during analysis. Principal component analysis (PCA) with 10 components at a resolution of 0.1 identified seven clusters. Dimension reduction plots, feature plots, violin plots and volcano plots were visualized by ggplot2 version 3.4.2 and EnhancedVolcano version 1.16.0. KEGG and GO analyses were performed using clusterProfiler version 4.7.1.003. *P*‐values were calculated by the Wilcoxon test with the ggpubr version 0.4.0 package.

### 4.12 Human Brain Samples

The study protocol involving human brain samples was approved by the Ethics Committee of Xijing Hospital (KY20202003). Human ischemic cortical samples were obtained from patients who met the following inclusion criteria: (1) aged 20 years or older; (2) required decompressive craniectomy and partial lobectomy for cerebral infarction at 18–26 h post ischemia; (3) provided informed consent. The exclusion criteria included: (1) the presence of malformation, tumor, infectious disease or other major brain disease; (2) patients deemed unsuitable for the study by the investigator. The ischemic cortical samples were fixed with 4% paraformaldehyde, embedded by Tissue‐Tek O.C.T. Compound (Sakura) and stored at −80°C until frozen section preparation for immunofluorescence staining.

### 4.13 Immunofluorescence Staining

Frozen serial sections of brain, liver, spleen, heart, lung, and kidney with a thickness of 12 µm were prepared using a cryostat (Leica). For immunofluorescence staining, slices were blocked with 3% bovine serum albumin and 0.3% Triton X‐100 in PBS for 1 h at room temperature. The sections were subjected to overnight incubation with primary antibodies, followed by a subsequent 2‐h incubation with secondary antibodies at room temperature. Details of the antibodies used are listed in Table S2. The sections were then mounted using Antifade Mounting Medium with DAPI (SouthernBiotech). Image acquisition was performed using an Olympus BX53 Microscope.

### 4.14 Hematoxylin and Eosin (H&E) Staining

Tissue samples were fixed in 4% paraformaldehyde, embedded in paraffin blocks, and sectioned at 5 µm thickness using a microtome (Leica). Hematoxylin and eosin Staining Kit (Beyotime Biotechnology Co., Ltd.) was used. Images were acquired using Olympus BX53 Microscope.

### 4.15 Evaluation of Blood‐Brain Barrier (BBB) Integrity

The assessment of blood‐brain barrier (BBB) integrity involved quantifying the extravasation of Evans blue (EB) dye (Meilun Biological Technology). Mice received a 2% EB solution in saline via tail vein injection at 1, 3, 5, and 7 dpi, at a dose of 4 mL kg^−1^. Transcranial perfusion with saline was conducted 2 h post‐EB injection to eliminate residual dye. Then brain tissues were weighed and images were captured. EB was extracted from brain tissues using methanamide solution (Sigma) and measured at 620 nm with a MULTISKAN FC microplate reader (Thermo Fisher Scientific). Results were expressed as the ratio of EB content in the injured ipsilateral hemisphere to that in the uninjured contralateral hemisphere.

### 4.16 Western Blotting

Samples were lysed by RIPA buffer, electrophoresed in 10% SDS‐PAGE gel, and then transferred to a polyvinylidene fluoride membrane. Blots were incubated with primary antibodies overnight at 4°C and followed by HRP‐tagged secondary antibodies. The blots were detected using Super ECL Plus (Applygen Technologies lnc) and quantified with ChemiDoc MP Imaging System (Bio‐Rad Laboratories, Inc.). Antibodies used were listed in the Table S2 and GAPDH was used as loading control.

### 4.17 Co‐Immunoprecipitation (CoIP) Assay

Nuclear proteins of BV2 cells were prepared using NE‐PER Nuclear and Cytoplasmic Extraction Reagents (Thermo Fisher Scientific) and Pierce IP Lysis Buffer (Thermo Fisher Scientific) according to the manufacturer's instructions. Equal amounts of protein were incubated with either C/EBPβ antibody or control IgG antibody at 4°C overnight, followed by incubating with Pierce Protein A/G Magnetic Beads (Thermo Fisher Scientific) for 1 h at room temperature. Beads were collected, washed five times with Pierce IP Lysis Buffer, and boiled at 95°C for 10 min to eluate protein. The eluted proteins were further identified by Western blotting and liquid chromatography‐tandem mass spectrometry (Orbitrap Exploris 480, Thermo Fisher Scientific) with the UniProtKB/Swiss‐Prot mouse database.

### 4.18 Chromatin Immunoprecipitation (ChIP) Assay

ChIP assays were conducted using the Pierce Magnetic ChIP Kit (Thermo Fisher Scientific) following the manufacturer's protocol. In brief, the nuclear fractions of BV2 cells were digested by micrococcal nuclease at 37°C for 15 min, followed by sonication using the JY92‐IIN Ultrasonic Homogenizer (Ningbo Scientz Biotechnology Co., Ltd) to yield chromatin fragments of 200 to 500 base pairs. Immunoprecipitation was performed overnight with antibodies against C/EBPβ, Cdkn1a, and control IgG. Complexes were then incubated with 25 µL chip‐grade protein A/G magnetic beads at 4°C for 2 h. Immunoprecipitated products were subjected to qRT‐PCR. Primers are detailed in Table S1.

### 4.19 Electrophoretic Mobility Shift Assay (EMSA)

Nuclear proteins were extracted from BV2 cells using NE‐PER Nuclear and Cytoplasmic Extraction Reagents (Thermo Fisher Scientific). Double‐stranded oligonucleotide probes targeting the binding sites of *C/EBPβ* were labeled with biotin or left unlabeled, referred to as biotinylated probes or cold probes. The sequence of probes was detailed in Table S1. EMSA was performed using the LightShift Chemiluminescent EMSA Kit (Thermo Fisher Scientific). Biotinylated probes (0.2 pmol) or corresponding cold probes (20 pmol) were incubated with 10 µg nuclear proteins at room temperature, followed by separation on native polyacrylamide gel. The gel was transferred to a positively charged nylon transfer membrane and crosslinked by ultraviolet irradiation. Biotinylated probes were detected using Streptavidin‐Horseradish Peroxidase Conjugate and Chemiluminescent Substrate, and visualized with the ChemiDoc MP Imaging System (Bio‐Rad Laboratories, Inc.).

### 4.20 Plasmid Construction

For overexpression, the coding sequence region of *C/EBPβ* (NM_009883.4) or *Cdkn1a* (NM_007669.5) was inserted into a pCDNA3.1(+) vector. For the plasmids used in the dual luciferase assay, the promoter regions were inserted into the pGL3‐Basic Vector (Promega Corporation). The insert fragments were positioned relative to the transcription start site (TSS) according to the University of California Santa Cruz (UCSC) Genome Browser Database. The selected promoter regions were as follows: *Il6* (‐450 bp to TSS), *Il1β* (−501 bp to TSS), *Cxcl2* (−2001 bp to TSS, −699 bp to TSS, −567 bp to TSS) and *Cxcl10* (−1452 bp to TSS, −366 bp to TSS, −252 bp to TSS).

### 4.21 Dual Luciferase Assay

For dual luciferase assay, pGL3‐Basic Vector, pRL‐TK Vector carrying Renilla luciferase (Promega Corporation), *C/EBPβ* overexpression plasmid and plasmids with insertion of selected promoter regions mentioned above were transfected into HEK293 cells using Lipofectamine 3000 (Thermo Fisher Scientific). Cell lysates were collected 48 h post transfection with Passive Lysis Buffer. Luciferase activity was measured using Dual‐Luciferase Reporter Assay System (Promega Corporation) and detected by Spark Multimode Microplate Reader (Tecan Group Ltd.).

### 4.22 Hematological and Biochemical Analyses

Blood samples were collected from the tail vein. Red blood cell (RBC) count, platelet (PLT) count, and white blood cell (RBC) count were measured for hematology assessment by using TEK‐II Mini hematology analyzer (Tecom Science Corporation, China). Alanine aminotransferase (ALT), aspartate aminotransferase (AST), alkaline phosphatase (ALP), blood urea nitrogen (BUN), creatinine (CR), uric acid (UA) were measured by using biochemical analyzer TBA‐40FR (Toshiba Medical Systems Corporation).

### 4.23 Behavioral Tests

Mice were randomly divided into three groups and pretrained for 4 days. Behavioral tests were performed and analyzed by two independent technicians who were blind to the group arrangement. For the Rotarod test, mice were placed in separate lanes on a rod of Rotarod system (NJKEWBIO). The speed of the rod was accelerated from 5 to 40 rpm over 180 s, followed by constant speed for the subsequent 3 min. The trial ended when a mouse fell off the rod and repeated thrice with a 30‐min interval between trials. The maximal distance was recorded.

For the foot fault test, the mice were allowed to walk on a horizontally positioned ladder with a rung spacing of 1.5 cm for 10 min. A step was classified as a foot fault if a slip or miss occurred. Foot faults of each limb were recorded, and the percentage of foot faults was calculated using the formula: the number of foot faults/(number of steps + number of foot faults) ×100.

For the cylinder test, the mice were placed in a transparent cylinder (height: 15 cm, diameter: 10 cm) and videoed for 10 min. Forelimb use is defined by the placement of the whole palm on the wall of the cylinder for body support during rearing. The forelimb asymmetric index was calculated as the ratio: (number of right forelimb uses − number of left forelimb uses) / (number of total forelimb uses) ×100.

For the latency to move test, a circle with a radius of 7 cm (approximately equivalent to one full body length) was drawn on a horizontal platform. The mouse was placed at the center of the circle, and the latency to move was defined as the time taken for the mouse to move out of the circle.

### 4.24 Statistical Analysis

Data are presented as mean ± SEM (standard error of the mean). The data were normalized to the control group for relative comparison, as detailed in each figure legend. Statistical analyses were performed using GraphPad Prism v8.0 (GraphPad Software Inc.). All primary data associated with the statistical analyses are provided in data file S1. The normality of the data was assessed using the Shapiro–Wilk test. For normally distributed data, statistical differences between two groups were determined using an unpaired two‐tailed Student's *t*‐test. For comparisons involving three or more groups, one‐way analysis of variance (ANOVA) followed by Tukey's multiple comparison tests was employed. Statistical significance was defined as *p < 0.05, **p < 0.01, and ***p < 0.001.

## Author Contributions

Conceptualization: Jialei Yang. Methodology: Jialei Yang, Shipo Wu, and Miao He. Investigation: Jialei Yang, Miao He, and Shipo Wu. Visualization: Jialei Yang and Miao He. Funding acquisition: Jialei Yang. Project administration and supervision: Jialei Yang and Shipo Wu. Writing – original draft: Jialei Yang. Writing – review and editing: Jialei Yang and Shipo Wu.

## Conflicts of Interest

The authors declare no conflicts of interest.

## Supporting information



Supporting Information

## Data Availability

The data that support the findings of this study are available in the supplementary material.

## References

[1] S. S. Martin , A. W. Aday , Z. I. Almarzooq , et al., “2024 Heart Disease and Stroke Statistics: A Report of US and Global Data From the American Heart Association,” Circle 149 (2024): e347. 10.1161/CIR.0000000000001209 PMC12146881 38264914

[2] E. Candelario‐Jalil , R. M. Dijkhuizen , and T. Magnus , “Neuroinflammation, Stroke, Blood‐Brain Barrier Dysfunction, and Imaging Modalities,” Stroke; A Journal of Cerebral Circulation 53 (2022): 1473. 10.1161/STROKEAHA.122.036946 PMC9038693 35387495

[3] C. López‐Otín , M. A. Blasco , L. Partridge , M. Serrano , and G. Kroemer , “Hallmarks of Aging: An Expanding Universe,” Cell 186 (2023): 243. 36599349 10.1016/j.cell.2022.11.001

[4] L. S. Melo Dos Santos , M. Trombetta‐Lima , B. J. L. Eggen , and M. Demaria , “Cellular Senescence in Brain Aging and Neurodegeneration,” Ageing Research Reviews 84 (2024): 101890. 10.1016/j.arr.2023.102141 38030088

[5] R. Di Micco , V. Krizhanovsky , D. Baker , and F. d'Adda di Fagagna , “Cellular Senescence in Ageing: From Mechanisms to Therapeutic Opportunities,” Nature Reviews Molecular Cell Biology 22 (2021): 75–95. 33328614 10.1038/s41580-020-00314-w PMC8344376

[6] I. K. Herrmann , M. J. A. Wood , and G. Fuhrmann , “Extracellular Vesicles as a Next‐Generation Drug Delivery Platform,” Nature Nanotechnology 16 (2021): 748–759. 10.1038/s41565-021-00931-2 34211166

[7] V. P. Chavda , G. Luo , T. K. R. Bezbaruah , et al., “Unveiling the Promise: Exosomes as Game‐Changers in Anti‐Infective Therapy,” Exploration 4 (2024): 20230139. 39439498 10.1002/EXP.20230139 PMC11491308

[8] J. Yang , Y. Li , S. Jiang , et al., “Engineered Brain‐Targeting Exosome for Reprogramming Immunosuppressive Microenvironment of Glioblastoma,” Exploration (2024): 20240039. 40395761 10.1002/EXP.20240039 PMC12087404

[9] J. Yang , X. Zhang , X. Chen , L. Wang , and G. Yang , “Exosome Mediated Delivery of miR‐124 Promotes Neurogenesis After Ischemia,” Molecular Therapy Nucleic Acids 7 (2017): 278–287. 28624203 10.1016/j.omtn.2017.04.010 PMC5415550

[10] J. Yang , S. Wu , L. Hou , et al., “Therapeutic Effects of Simultaneous Delivery of Nerve Growth Factor mRNA and Protein via Exosomes on Cerebral Ischemia,” Molecular Therapy Nucleic Acids 21 (2020): 512–522. 32682291 10.1016/j.omtn.2020.06.013 PMC7365960

[11] Z. G. Zhang , B. Buller , and M. Chopp , “Exosomes — Beyond Stem Cells for Restorative Therapy in Stroke and Neurological Injury,” Nature Reviews Neurology 15 (2019): 193–203. 30700824 10.1038/s41582-018-0126-4

[12] S. Kim , W. Lee , H. Jo , et al., “The Antioxidant Enzyme Peroxiredoxin‐1 Controls Stroke‐Associated Microglia Against Acute Ischemic Stroke,” Redox Biology 54 (2022): 102347. 35688114 10.1016/j.redox.2022.102347 PMC9184746

[13] M. Pulido‐Salgado , J. M. Vidal‐Taboada , and J. Saura , “C/EBPβ and C/EBPδ Transcription Factors: Basic Biology and Roles in the CNS,” Progress in Neurobiology 132 (2015): 1–33. 26143335 10.1016/j.pneurobio.2015.06.003

[14] C. Lee and C.‐H. Huang , “LASAGNA‐Search 2.0: Integrated Transcription Factor Binding Site Search and Visualization in a Browser,” Bioinformatics 30 (2014): 1923–1925. 24578403 10.1093/bioinformatics/btu115

[15] O. Fornes , J. A. Castro‐Mondragon , A. Khan , et al., “JASPAR 2020: Update of the Open‐Access Database of Transcription Factor Binding Profiles,” Nucleic Acids Research 48 (2020): D87–D92. 31701148 10.1093/nar/gkz1001 PMC7145627

[16] T. Terashima , N. Ogawa , Y. Nakae , et al., “Gene Therapy for Neuropathic Pain Through siRNA‐IRF5 Gene Delivery With Homing Peptides to Microglia,” Molecular Therapy Nucleic Acids 11 (2018): 203–215. 29858055 10.1016/j.omtn.2018.02.007 PMC5992689

[17] T. Yamashita , Y. Takahashi , and Y. Takakura , “Possibility of Exosome‐Based Therapeutics and Challenges in Production of Exosomes Eligible for Therapeutic Application,” Biological and Pharmaceutical Bulletin 41 (2018): 835. 29863072 10.1248/bpb.b18-00133

[18] E. Tzng , N. Bayardo , and P. C. Yang , “Current Challenges Surrounding Exosome Treatments,” Extracellular Vesicle 2 (2023): 100023. 40027080 10.1016/j.vesic.2023.100023 PMC11870656

[19] R. L. Cohn , N. S. Gasek , G. A. Kuchel , and M. Xu , “The Heterogeneity of Cellular Senescence: Insights at the Single‐Cell Level,” Trends in Cell Biology 33 (2023): 9–17. 35599179 10.1016/j.tcb.2022.04.011 PMC9812642

[20] J. Baixauli‐Martín , A. Aliena‐Valero , M. Castelló‐Ruiz , et al., “Brain Cell Senescence: A New Therapeutic Target for the Acute Treatment of Ischemic Stroke,” Journal of Neuropathology and Experimental Neurology 81 (2022): 614–620. 35763058 10.1093/jnen/nlac048

[21] C. Torres‐Querol , P. Torres , N. Vidal , M. Portero‐Otín , G. Arque , and F. Purroy , “Acute Ischemic Stroke Triggers a Cellular Senescence‐Associated Secretory Phenotype,” Scientific Reports 11 (2021): 15752. 34344977 10.1038/s41598-021-95344-5 PMC8333348

[22] S. Chaib , T. Tchkonia , and J. L. Kirkland , “Cellular Senescence and Senolytics: The Path to the Clinic,” Nature Medicine 28 (2022): 1556–1568. 10.1038/s41591-022-01923-y PMC9599677 35953721

[23] A. Papadopoulos , K. Palaiopanos , H. Björkbacka , et al., “Circulating Interleukin‐6 Levels and Incident Ischemic Stroke,” Neurology 98 (2022): e1002. 34969940 10.1212/WNL.0000000000013274 PMC8967391

[24] S. S. Shaftel , T. J. Carlson , J. A. Olschowka , S. Kyrkanides , S. B. Matousek , and M. K. O'Banion , “Chronic Interleukin‐1β Expression in Mouse Brain Leads to Leukocyte Infiltration and Neutrophil‐Independent Blood–Brain Barrier Permeability Without Overt Neurodegeneration,” Journal of Neuroscience 27 (2007): 9301–9309. 17728444 10.1523/JNEUROSCI.1418-07.2007 PMC6673122

[25] K. De Filippo , A. Dudeck , M. Hasenberg , et al., “Mast Cell and Macrophage Chemokines CXCL1/CXCL2 Control the Early Stage of Neutrophil Recruitment During Tissue Inflammation,” Blood 121 (2013): 4930–4937. 23645836 10.1182/blood-2013-02-486217

[26] Q. Chai , R. She , Y. Huang , and Z. F. Fu , “Expression of Neuronal CXCL10 Induced by Rabies Virus Infection Initiates Infiltration of Inflammatory Cells, Production of Chemokines and Cytokines, and Enhancement of Blood‐Brain Barrier Permeability,” Journal of Virology 89 (2015): 870. 25339777 10.1128/JVI.02154-14 PMC4301165

[27] T. Kuilman , C. Michaloglou , L. C. W. Vredeveld , et al., “Oncogene‐Induced Senescence Relayed by an Interleukin‐Dependent Inflammatory Network,” Cell 133 (2008): 1019–1031. 18555778 10.1016/j.cell.2008.03.039

[28] C. Basak , S. K. Pathak , A. Bhattacharyya , D. Mandal , S. Pathak , and M. Kundu , “NF‐κB‐ and C/EBPβ‐driven Interleukin‐1β Gene Expression and PAK1‐Mediated Caspase‐1 Activation Play Essential Roles in Interleukin‐1β Release From Helicobacter Pylori Lipopolysaccharide‐Stimulated Macrophages,” Journal of Biological Chemistry 280 (2005): 4279–4288. 15561713 10.1074/jbc.M412820200

[29] E. R. Westin , A. Khodadadi‐Jamayran , L. K. Pham , M. L. Tung , and F. D. Goldman , “CRISPR Screen Identifies CEBPB as Contributor to Dyskeratosis Congenita Fibroblast Senescence via Augmented Inflammatory Gene Response,” G3 Genes|Genomes|Genetics 13 (2023): jkad207. 37717172 10.1093/g3journal/jkad207 PMC10627266

[30] J. C. L. Spurrell , S. Wiehler , R. S. Zaheer , S. P. Sanders , and D. Proud , “Human Airway Epithelial Cells Produce IP‐10 (CXCL10) In Vitro and In Vivo Upon Rhinovirus Infection,” American Journal of Physiology. Lung Cellular and Molecular Physiology 289 (2005): L85–L95. 15764644 10.1152/ajplung.00397.2004

[31] L. Zhang , L. E. Pitcher , M. J. Yousefzadeh , L. J. Niedernhofer , P. D. Robbins , and Y. Zhu , “Cellular Senescence: A Key Therapeutic Target in Aging and Diseases,” Journal of Clinical Investigation 132 (2022): e158450. 35912854 10.1172/JCI158450 PMC9337830

[32] Z. Erana‐Perez , M. Igartua , E. Santos‐Vizcaino , and R. M. Hernandez , “Genetically Engineered Loaded Extracellular Vesicles for Drug Delivery,” Trends in Pharmacological Sciences 45 (2024): 350–365. 38508958 10.1016/j.tips.2024.02.006

[33] M. M. Gonzales , V. R. Garbarino , T. F. Kautz , et al., “Senolytic Therapy in Mild Alzheimer's Disease: A Phase 1 Feasibility Trial,” Nature Medicine 29 (2023): 2481–2488. 10.1038/s41591-023-02543-w PMC10875739 37679434

[34] A. Bardestani , S. Ebrahimpour , A. Esmaeili , and A. Esmaeili , “Quercetin Attenuates Neurotoxicity Induced by Iron Oxide Nanoparticles,” Journal of Nanobiotechnology 19 (2021): 327. 34663344 10.1186/s12951-021-01059-0 PMC8522232

[35] L. G. Costa , J. M. Garrick , P. J. Roquè , and C. Pellacani , “Mechanisms of Neuroprotection by Quercetin: Counteracting Oxidative Stress and More,” Oxidative Medicine and Cellular Longevity 2016 (2016): 2986796. 26904161 10.1155/2016/2986796 PMC4745323

[36] G. M. Keating , “Dasatinib: A Review in Chronic Myeloid Leukaemia and Ph+ Acute Lymphoblastic Leukaemia,” Drugs 77 (2017): 85–96. 28032244 10.1007/s40265-016-0677-x

[37] F. Meiners , B. Hinz , L. Boeckmann , et al., “Computational Identification of Natural Senotherapeutic Compounds that Mimic Dasatinib Based on Gene Expression Data,” Scientific Reports 14 (2024): 6286. 38491064 10.1038/s41598-024-55870-4 PMC10943199

[38] A. Duong , G. Parmar , A. M. Kirkham , D. Burger , and D. S. Allan , “Registered Clinical Trials Investigating Treatment With Cell‐Derived Extracellular Vesicles: A Scoping Review,” Cytotherapy 25, no. 9 (2023): 939–945. 37191614 10.1016/j.jcyt.2023.04.007

[39] J. Dumont , D. Euwart , B. Mei , S. Estes , and R. Kshirsagar , “Human Cell Lines for Biopharmaceutical Manufacturing: History, Status, and Future Perspectives,” Critical Reviews in Biotechnology 36, no. 6 (2016): 1110–1122. 26383226 10.3109/07388551.2015.1084266 PMC5152558

[40] J.‐C. D. Schwartz , X. Zhang , A. A. Fedorov , S. G. Nathenson , and S. C. Almo , “Structural Basis for Co‐Stimulation by the Human CTLA‐4/B7‐2 Complex,” Nature 410 (2001): 604–608. 11279501 10.1038/35069112

[41] G. J. Freeman , G. S. Gray , C. D. Gimmi , et al., “Structure, Expression, and T Cell Costimulatory Activity of the Murine Homologue of the Human B Lymphocyte Activation Antigen B7,” Journal of Experimental Medicine 174, no. 3 (1991): 625–631. 1714935 10.1084/jem.174.3.625 PMC2118924

[42] D. J. Lenschow , G. H. Su , L. A. Zuckerman , et al., “Expression and Functional Significance of an Additional Ligand for CTLA‐4,” Proceedings National Academy of Science USA 90 (1993): 11054–11058. 10.1073/pnas.90.23.11054 PMC47920 7504292

[43] R. J. Greenwald , G. J. Freeman , and A. H. Sharpe , “The B7 Family Revisited,” Annual Review of Immunology 23 (2005): 515–548. 10.1146/annurev.immunol.23.021704.115611 15771580

[44] X. Zhao , J. Wang , Y. Deng , et al., “Quercetin as a Protective Agent for Liver Diseases: A Comprehensive Descriptive Review of the Molecular Mechanism,” Phytotherapy Research 35, no. 9 (2021): 4727–4747. 34159683 10.1002/ptr.7104

[45] N. Li , C. Cui , J. Xu , M. Mi , J. Wang , and Y. Qin , “Quercetin Intervention Reduced Hepatic Fat Deposition in Patients With Nonalcoholic Fatty Liver Disease: A Randomized, Double‐Blind, Placebo‐Controlled Crossover Clinical Trial,” American Journal of Clinical Nutrition 120, no. 3 (2024): 507–517. 39032786 10.1016/j.ajcnut.2024.07.013

