# Supplementary material for: Engineered Exosome‐Based Senolytic Therapy Alleviates Stroke by Targeting p21+CD86+ Microglia
Source: Exploration (Beijing). 2025 Mar 6;5(3):20240349. doi: 10.1002/EXP.20240349 (PMC12199405; doi:10.1002/EXP.20240349)
Supplement: Supplementary file 1 — Supporting Information [file EXP2-5-20240349-s001.docx]

**Supporting Information**

**Engineered exosome-based senolytic therapy alleviates stroke by targeting p21^+^CD86^+^ microglia**

**Authors:** Jialei Yang^1*^, Shipo Wu^2^, Miao He^1^.

**Author address:**

^1^ Department of Neurology, China National Clinical Research Center for Neurological Diseases, Beijing Tiantan Hospital, Capital Medical University; Beijing, China.

^2^ Laboratory of Advanced Biotechnology, Beijing Institute of Biotechnology; Beijing, China.

The authors claim no conflicts of interests.

*Correspondence should be addressed to Jialei Yang.

**Supplementary Figures**

**
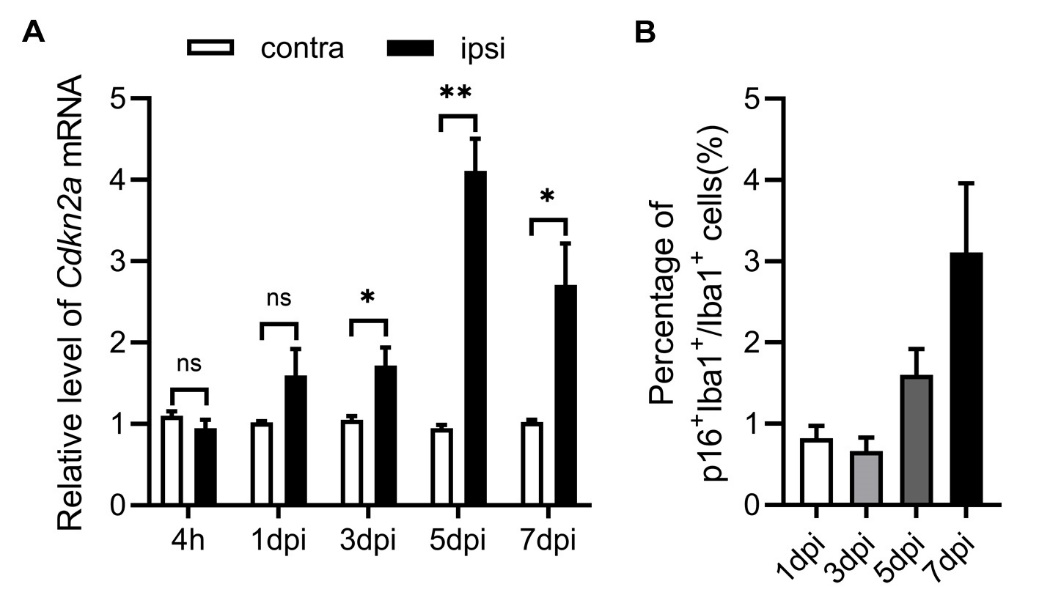
**

**Figure S1. The expression of p16 in ischemic regions.**

(**A**) *Cdkn2a* mRNA levels in contralateral (contra) and ipsilateral (ipsi) regions at 1, 3, 5, and 7 days post-ischemia (dpi) in a mouse ischemic model. (**B**) Quantification of the percentage of p16/Iba double-positive cells. Data are expressed as mean ± SEM, with n = 3 per group. Data were analyzed by an unpaired two-tailed Student’s t-test. **P* < 0.05; ***P* < 0.01; ns, not significant.


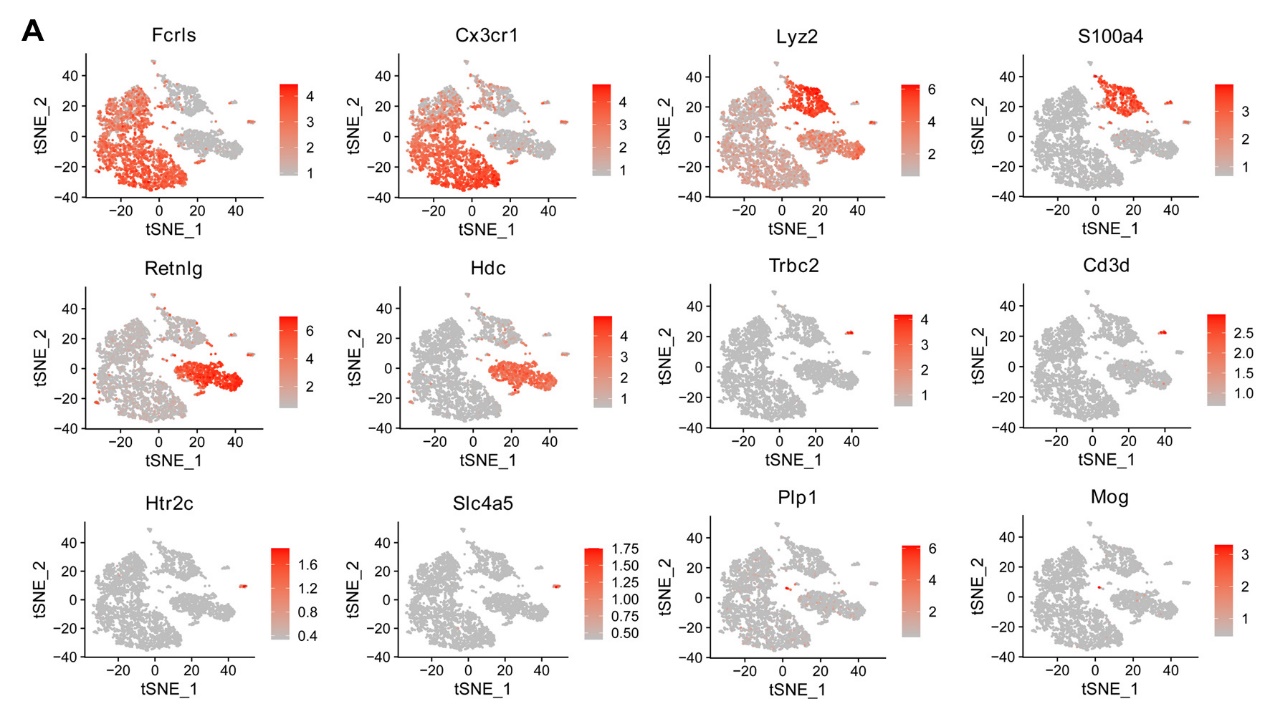


**Figure S2. Representative markers for the identification of each cell cluster.**

(**A**) t-SNE plots showing representative markers for each cell cluster in the ischemic brain. Microglia (*Fcrls, Cx3cr1*), macrophage (*Lyz2, S100a4*), neutrophil (*Retnlg, Hdc*), T cells (*Trbc2, Cd3d*), choroid plexus cells (*Htr2c, Slc4a5*) and oligodendrocytes (*Plp1, Mog*).

**
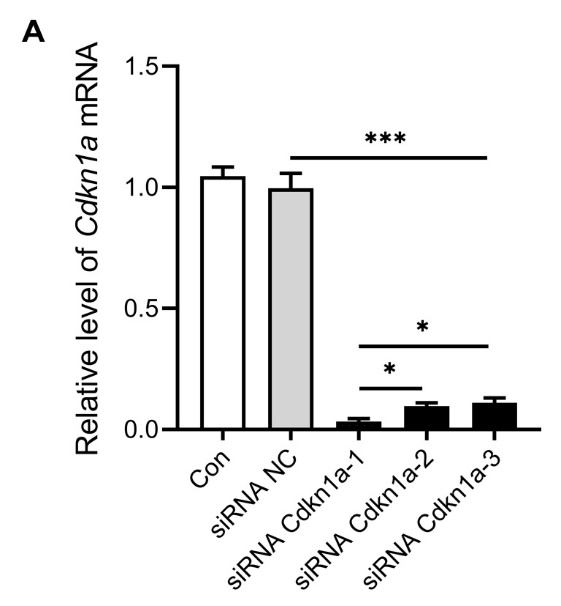
**

**Figure S3. Knockdown efficiency of *Cdkn1a* using siRNA in BV2 Cells.**

(**A**) qRT-PCR analysis showing the relative mRNA expression levels of *Cdkn1a* in BV2 cells, comparing untreated control (Con) and cells treated with siRNA negative control (siRNA NC), and three different siRNAs targeting *Cdkn1a* (siRNA Cdkn1a-1, siRNA Cdkn1a-2, and siRNA Cdkn1a-3). Data are expressed as mean ± SEM, with n = 3 per group. Statistical analysis was performed using one-way ANOVA followed by Tukey's post-hoc test. **P* < 0.05; ****P* < 0.001.


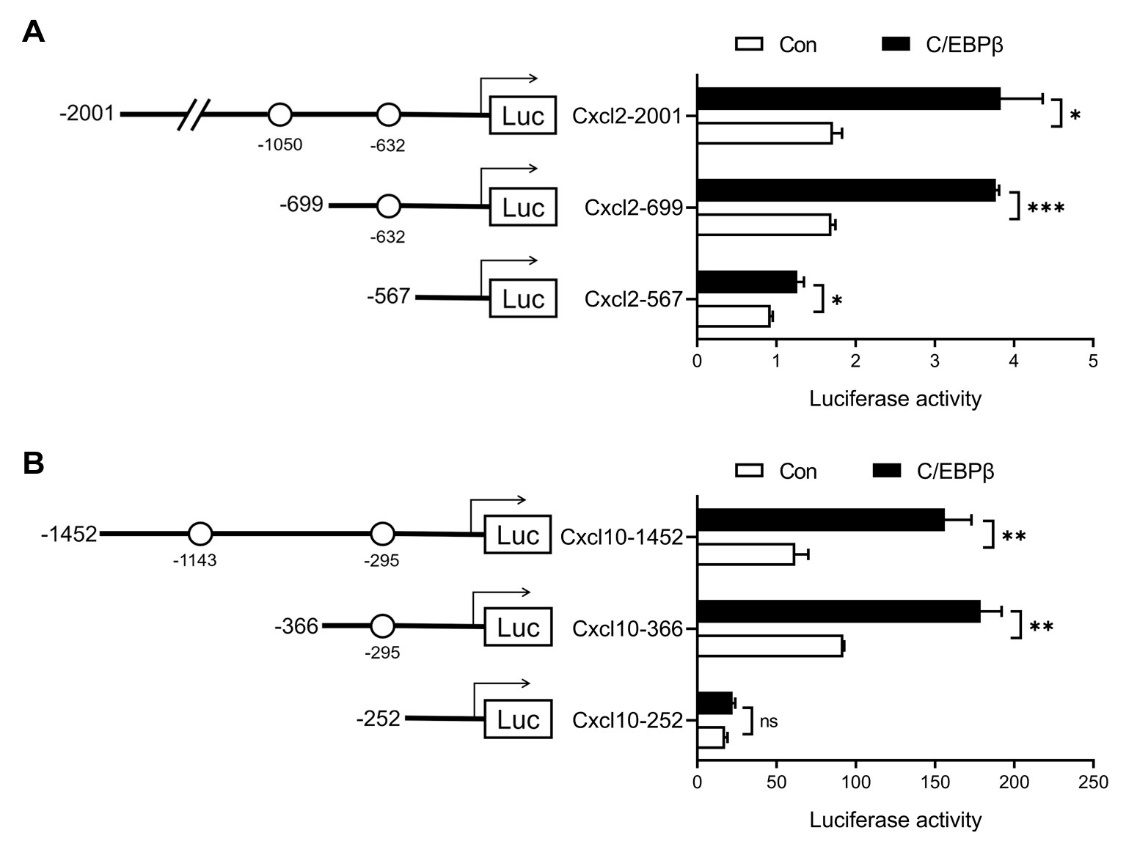


**Figure S4.** **Analysis of C/EBPβ transcriptional activation of the *Cxcl2* and *Cxcl10* promoter using luciferase reporter assays**

(**A-B**) Schematic representation of the *Cxcl2* and *Cxcl10* promoter constructs used in the luciferase assays. Luciferase activity in HEK293 cells co-transfected with the C/EBPβ overexpression plasmid and the various *Cxcl2* and *Cxcl10* promoter reporter constructs. The luciferase activity demonstrates significant transcriptional activation of the *Cxcl2* promoter (-699bp to -567bp), and the *Cxcl10* promoter (-366bp to -252bp) under C/EBPβ regulation. The circle represents the predicted binding site of C/EBPβ. Luc represents the firefly luciferase. Data are expressed as mean ± SEM, with n = 3 per group. Data were analyzed by an unpaired two-tailed Student’s t-test. **P* < 0.05; ***P* < 0.01; ****P*<0.001; ns, not significant.


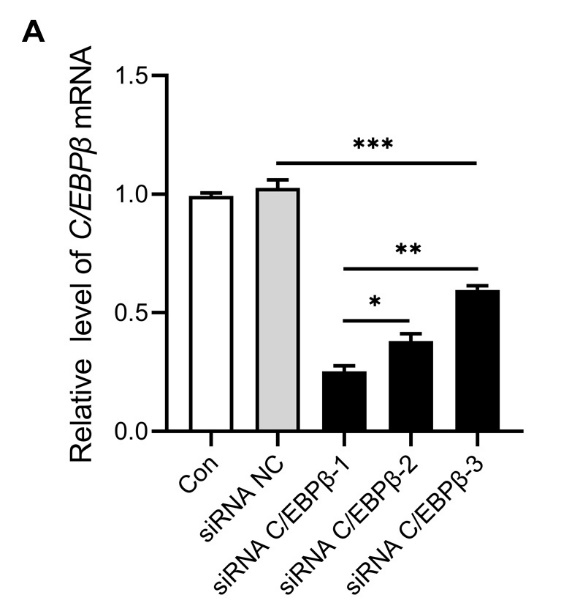


**Figure S5. Knockdown efficiency of *C/EBPβ* using siRNA in BV2 Cells.**

(**A**) qRT-PCR analysis showing the relative mRNA expression levels of *C/EBPβ* in BV2 cells, comparing untreated control (Con) and cells treated with siRNA negative control (siRNA NC), and three different siRNAs targeting *C/EBPβ* (siRNA C/EBPβ-1, siRNA C/EBPβ-2, and siRNA C/EBPβ-3). Data are expressed as mean ± SEM, with n = 3 per group. Statistical analysis was performed using one-way ANOVA followed by Tukey's post-hoc test. **P* < 0.05; ***P* < 0.01; ****P* < 0.001.


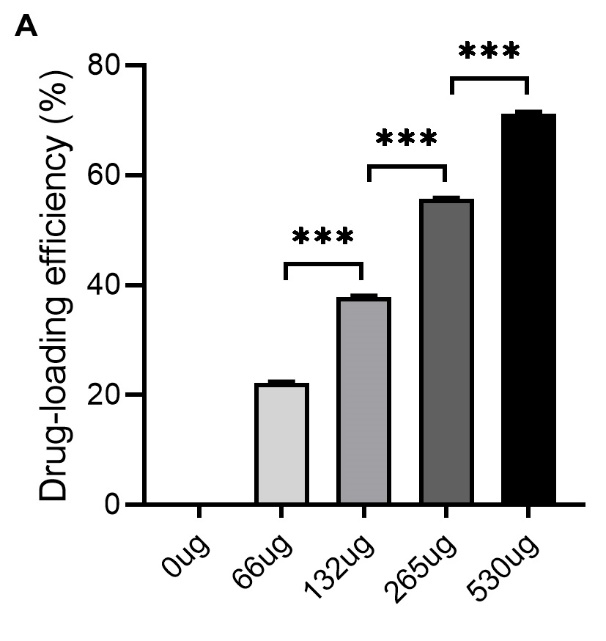


**Figure S6.** **Drug-loading efficiency.**

(**A**) Drug-loading efficiency of quercetin within the exosomes at different quercetin concentrations (0 µg, 66 µg, 132 µg, 265 µg, 530 µg). Data are expressed as mean ± SEM, with n = 3 per group. Data were analyzed by an unpaired two-tailed Student’s t-test. ****P*<0.001.


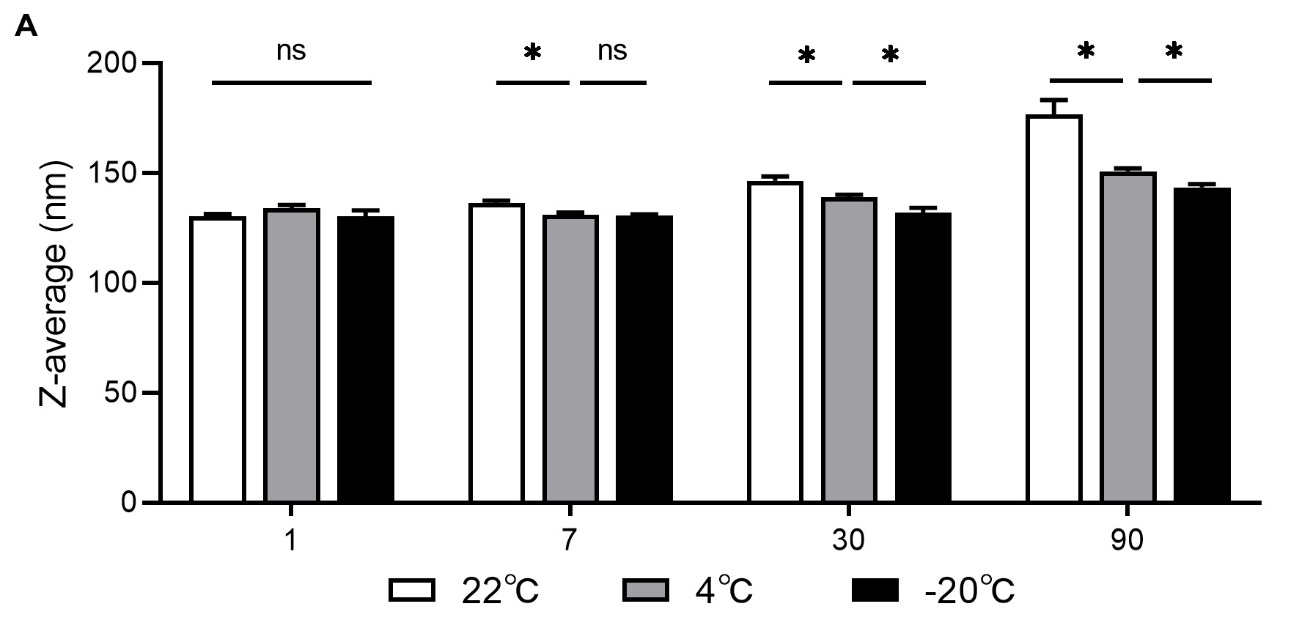


**Figure S7.** **Z-average size of Que@micro-Exo.**

(**A**) Z-average size of Que@micro-Exo at 22℃, 4℃, and -20℃ for 1, 7, 30, and 90 days, respectively. Data are expressed as mean ± SEM, with n = 3 per group. Statistical analysis was performed using one-way ANOVA followed by Tukey's post-hoc test. **P* < 0.05; ns, not significant.


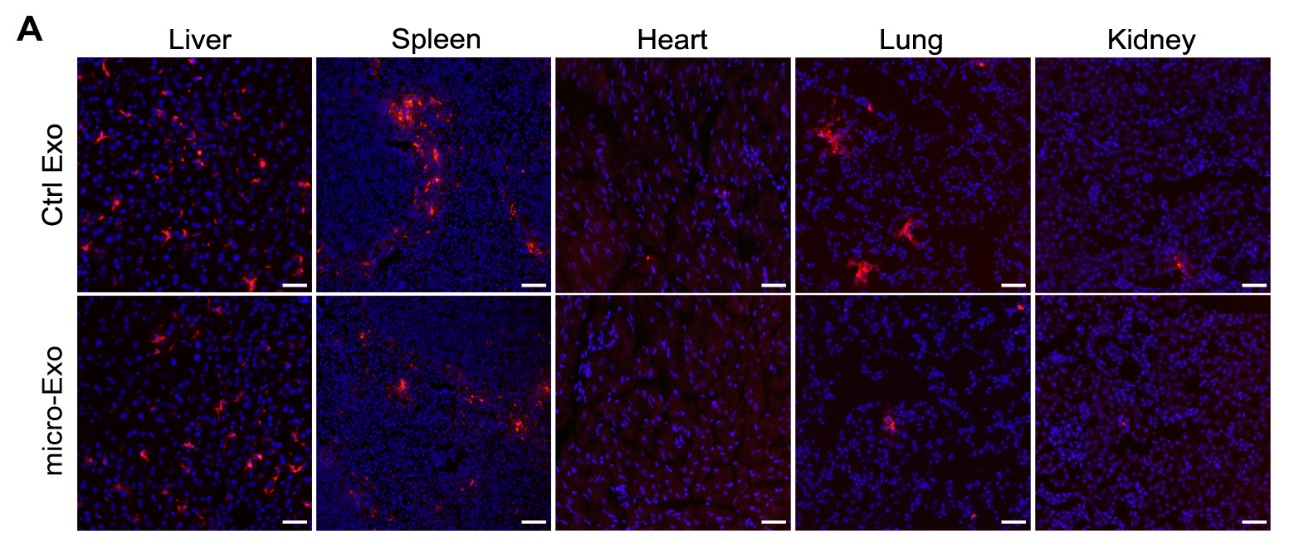


**Figure S8. Distribution of DiI-labeled exosomes in different tissues.**

(**A**) Representative immunofluorescence images showing the distribution of DiI-labeled control exosomes (Ctrl Exo) and microglia-targeted exosomes (micro-Exo) in liver, spleen, heart, lung and kidney. Nuclei are stained with DAPI (blue). Scale bar =50 μm.


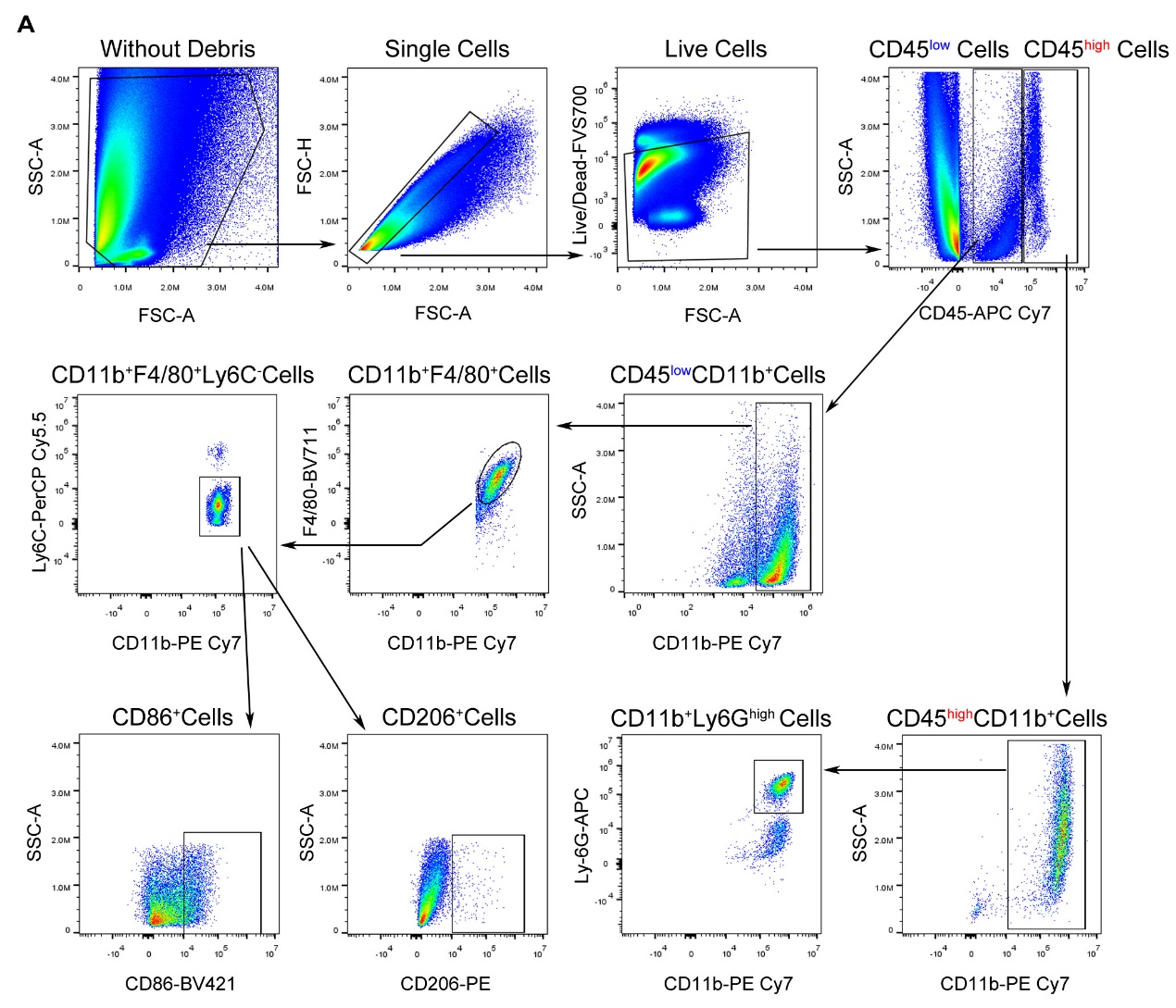


**Figure S9. Flow cytometry gating strategy.**

(**A**) The flow cytometry gating strategy used to identify cell populations. CD45^+^ for immune cells, CD11b^+^F4/80^+^Ly6C^−^CD86^+^ for M1 microglia, CD11b^+^F4/80^+^Ly6C^−^CD206^+^ for M2 microglia, and CD11b^+^Ly6G^high^ for neutrophils.

**Supplementary Tables**

**Table S1. Primers, siRNAs and probes used in this study.**

| **qRT-PCR** | **Forward Primer** | **Reverse Primer** |
| --- | --- | --- |
| *Cdkn1a* | CTTGCACTCTGGTGTCTGAG | GCACTTCAGGGTTTTCTCTTG |
| *Cdkn2a* | GTGCGATATTTGCGTTCCG | TCTGCTCTTGGGATTGGC |
| *Il6* | CAAAGCCAGAGTCCTTCAGAG | GTCCTTAGCCACTCCTTCTG |
| *Il1β* | ACGGACCCCAAAAGATGAAG | TTCTCCACAGCCACAATGAG |
| *Cxcl2* | AATGCCTGAAGACCCTGC | TTTTGACCGCCCTTGAGAG |
| *Cxcl10* | TCAGCACCATGAACCCAAG | CTATGGCCCTCATTCTCACTG |
| *Actb* | GTGCTATGTTGCTCTAGACTTCG | ATGCCACAGGATTCCATACC |
| **ChIP qRT-PCR** | **Forward Primer** | **Reverse Primer** |
| *Il6* | CCCCTTCCTAGTTGTGATTC | AAATCTTTGTTGGAGGGTGG |
| *Il1β* | TCCAACTTGTTTTCCCTCCCTT | ATCTGCCACCCCTTGACTTC |
| *Cxcl2* | CACTGGAAGAGCCTCGGAAG | CGTCTGCGTAAGTACACCGT |
| *Cxcl10* | GGGAAGTCCCCTGTAAACCG | TTCTGCAAGGCACTGCATCT |
| **EMSA probe** | **Forward strand** | **Reverse strand** |
| *Il6* | CGACGTCACATTGTGCAATCTTAATA | TATTAAGATTGCACAATGTGACGTCG |
| *Il1β* | CAGTTTTGTTGTGAAATCAGTTAACC | GGTTAACTGATTTCACAACAAAACTG |
| *Cxcl2* | TATATGAGATTACAACACTTTGTCCT | AGGACAAAGTGTTGTAATCTCATATA |
| *Cxcl10* | GCTTTGGAAAGTGAAACTTACCTCACTC | GAGTGAGGTAAGTTTCACTTTCCAAAGC |
| **siRNA** | **Sense strand** | **Antisense strand** |
| siRNA-*C/EBPβ*-1 | UGCAAUCCGGAUCAAACGUTT | ACGUUUGAUCCGGAUUGCATT |
| siRNA-*C/EBPβ*-2 | CCCUGCGGAACUUGUUCAAGCAGCU | AGCUGCUUGAACAAGUUCCGCAGGG |
| siRNA-*C/EBPβ*-3 | GGAACUUGUUCAAGCAGCUTT | AGCUGCUUGAACAAGUUCCTT |
| siRNA-*Cdkn1a*-1 | AUCACUCCAAGCGCAGAUUTT | AAUCUGCGCUUGGAGUGAUAG |
| siRNA-*Cdkn1a*-2 | CCAGCCUGACAGAUUUCUATT | UAGAAAUCUGUCAGGCUGGUC |
| siRNA-*Cdkn1a*-3 | GUCUCCAAACUUAAAGUUATT | UAACUUUAAGUUUGGAGACTT |
| siRNA-NC | UUCUCCGAACGUGUCACGUTT | ACGUGACACGUUCGGAGAATT |

**Table S2. List of antibodies used in this study.**

| **Antibodies** | **Vendor** | **RRID** |
| --- | --- | --- |
| Rabbit anti-C/EBPβ | Abcam | AB_726796 |
| Mouse anti-C/EBPβ | BioLegend | AB_315675 |
| Rabbit anti-p21 Waf1/Cip1 | Cell Signaling Technology | AB_2892063 |
| Mouse anti-p21 | Santa Cruz Biotechnology | AB_628073 |
| GAPDH (D16H11) XP Rabbit mAb | Cell Signaling Technology | AB_10622025 |
| Normal rabbit IgG antibody | Cell Signaling Technology | AB_1031062 |
| Mouse mAb IgG2b Isotype Control antibody | Cell Signaling Technology | AB_2799435 |
| Anti-rabbit IgG, HRP-linked | Cell Signaling Technology | AB_2099233 |
| Anti-mouse IgG, HRP-linked | Cell Signaling Technology | AB_330924 |
| Rabbit anti-Lamp2b | Abcam | AB_2940865 |
| Rabbit anti-CD63 | Abcam | AB_2754982 |
| Mouse anti-Tsg101 | Abcam | AB_306450 |
| Mouse anti-Alix | Cell Signaling Technology | AB_2299455 |
| Rabbit anti-GM130 | Abcam | AB_880266 |
| APC-Cy^TM^7 Rat Anti-Mouse CD45 | BD Biosciences | AB_2869637 |
| FITC Hamster Anti-Mouse CD3e | BD Biosciences | AB_396774 |
| BV605 Rat Anti-Mouse CD4 | BD Biosciences | AB_394594 |
| BV510 Rat Anti-Mouse CD8a | BD Biosciences | AB_2687549 |
| PE-Cy^TM^7 Rat Anti-CD11b | BD Biosciences | AB_2687548 |
| APC Rat Anti-Mouse Ly-6G | BD Biosciences | AB_394491 |
| BV711 Rat Anti-Mouse F4/80 | BD Biosciences | AB_1727560 |
| PerCP-Cy^TM^5.5 Rat Anti-Mouse Ly-6C | BD Biosciences | AB_2734769 |
| BV421 Rat Anti-Mouse CD86 | BD Biosciences | AB_1727558 |
| PE Rat Anti-Mouse CD206 | BD Biosciences | AB_2738663 |
| Goat anti-Iba1 | Abcam | AB_2224402 |
| Goat anti-CD206 | R and D Systems | AB_2063012 |
| Rat anti-CD86 | Thermo Fisher Scientific | AB_467368 |
| Rabbit anti-p21 (react with mice) | Abcam | AB_2734729 |
| Rabbit anti-p21 (react with human) | Thermo Fisher Scientific | AB_2914399 |
